# SARS-CoV-2 infection and risk of subsequent demyelinating diseases: national register–based cohort study

**DOI:** 10.1093/braincomms/fcae406

**Published:** 2024-11-29

**Authors:** Scott Montgomery, Snieguole Vingeliene, Huiqi Li, Helena Backman, Ruzan Udumyan, Johan Jendeberg, Gunlög Rasmussen, Martin Sundqvist, Katja Fall, Ayako Hiyoshi, Fredrik Nyberg

**Affiliations:** Clinical Epidemiology and Biostatistics, School of Medical Sciences, Faculty of Medicine and Health, Örebro University, 703 62 Örebro, Sweden; Division of Clinical Epidemiology, Department of Medicine, Solna, Karolinska Institutet, 171 77 Stockholm, Sweden; Department of Epidemiology and Public Health, University College London, London WC1E 7HB, UK; Clinical Epidemiology and Biostatistics, School of Medical Sciences, Faculty of Medicine and Health, Örebro University, 703 62 Örebro, Sweden; School of Public Health and Community Medicine, Institute of Medicine, Sahlgrenska Academy, University of Gothenburg, 405 30 Gothenburg, Sweden; Department of Obstetrics and Gynaecology, Faculty of Medicine and Health, Örebro University, 701 85 Örebro, Sweden; Clinical Epidemiology and Biostatistics, School of Medical Sciences, Faculty of Medicine and Health, Örebro University, 703 62 Örebro, Sweden; Department of Radiology, Faculty of Medicine and Health, Örebro University, 701 85 Örebro, Sweden; Department of Infectious Diseases, School of Medical Sciences, Faculty of Medicine and Health, Örebro University, 701 85 Örebro, Sweden; Department of Laboratory Medicine, Clinical Microbiology, Faculty of Medicine and Health, Örebro University, 701 85 Örebro, Sweden; Clinical Epidemiology and Biostatistics, School of Medical Sciences, Faculty of Medicine and Health, Örebro University, 703 62 Örebro, Sweden; The Institute of Environmental Medicine, Karolinska Institutet, 171 77 Stockholm, Sweden; Clinical Epidemiology and Biostatistics, School of Medical Sciences, Faculty of Medicine and Health, Örebro University, 703 62 Örebro, Sweden; School of Public Health and Community Medicine, Institute of Medicine, Sahlgrenska Academy, University of Gothenburg, 405 30 Gothenburg, Sweden

**Keywords:** SARS-CoV-2, multiple sclerosis, demyelinating disease

## Abstract

Demyelinating diseases including multiple sclerosis are associated with prior infectious exposures, so we assessed whether SARS-CoV-2 infection is associated with subsequent diagnoses of non-multiple sclerosis demyelinating diseases and multiple sclerosis. All residents of Sweden aged 3–100 years were followed between 1 January 2020 and 30 November 2022, excluding those with demyelinating disease prior to 2020, comprising 9 959 818 individuals divided into uninfected and those who were infected were categorized into those with and without hospital admission for the infection as a marker of infection severity. Cox regression assessed the risk of two separate outcomes: hospital diagnosed non-multiple sclerosis demyelinating diseases of the CNS and multiple sclerosis. The exposures were modelled as time-varying covariates (uninfected, infection without hospital admission and infected with hospital admission). Hospital admission for COVID-19 was associated with raised risk of subsequent non-multiple sclerosis demyelinating disease, but only 12 individuals had this outcome among the exposed, and of those, 7 has an unspecified demyelinating disease diagnosis. Rates per 100 000 person-years (and 95% confidence intervals) were 3.8 (3.6–4.1) among those without a COVID-19 diagnosis and 9.0 (5.1–15.9) among those admitted to hospital for COVID-19, with an adjusted hazard ratio and (and 95% confidence interval) of 2.35 (1.32–4.18, *P* = 0.004). Equivalent associations with multiple sclerosis (28 individuals had this outcome among the exposed) were rates of 9.5 (9.1–9.9) and 21.0 (14.5–30.5) and an adjusted hazard ratio of 2.48 (1.70–3.61, *P* < 0.001). Only a small number of non-multiple sclerosis demyelinating disease diagnoses were associated with hospital admission for COVID-19, and while the number with multiple sclerosis was somewhat higher, longer duration of follow-up will assist in identifying whether the associations are causal or due to shared susceptibility or surveillance bias, as these diseases can have long asymptomatic and prodromal phases.

## Teaser

Montgomery *et al*. report that hospital admission for COVID-19 was associated with raised risks of subsequent non-multiple sclerosis demyelinating diseases of the CNS and multiple sclerosis. Longer term follow-up is required to determine if the associations are causal or have other explanations, such as shared susceptibility or increased contact with healthcare.

## Graphical Abstract

**Graphical Abstract:**
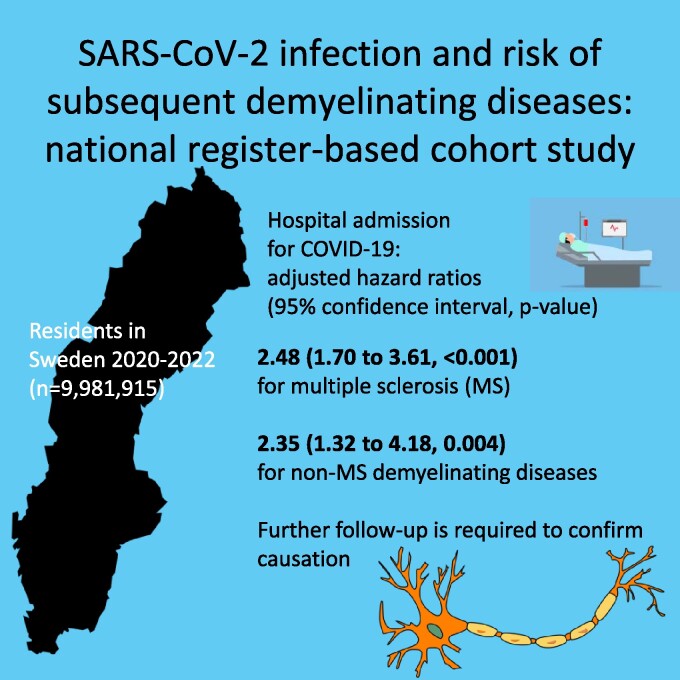

## Introduction

There is some limited evidence to suggest that SARS-CoV-2 infection may be associated with demyelinating diseases of the CNS.^1,2^ Given SARS-CoV-2 infection can have consequences for the CNS, including autoimmune disorders,^3^ how likely is it that it increases risk of demyelinating diseases of the CNS? This is possibly consistent with the association of some types of viral infections, particularly due to Epstein–Barr virus, and risk of subsequent multiple sclerosis.^4-6^ However, as there can be an extended asymptomatic/prodromal period in multiple sclerosis and there may even be a 10–20-year duration between an acute triggering infection and a multiple sclerosis diagnosis,^6-8^ it may be premature to assess full associations with multiple sclerosis.

In excess of 30% of patients with more severe COVID-19 have CNS lesions^9^ and a case-series study of three patients, including one meeting multiple sclerosis criteria, suggests the infection could be implicated in causing inflammatory demyelinating disease.^10^ In addition to multiple sclerosis, there are two further categories of CNS demyelinating disease in the Swedish version of the International Classification of Diseases 10 (ICD-10). The first is ‘other acute disseminated demyelination’ that includes neuromyelitis optica spectrum disorders, which involve optic nerve inflammation, and clinically isolated syndrome, which is a first neurological episode that may represent onset of multiple sclerosis in some individuals. The second category is ‘other demyelinating diseases of the CNS’, which includes diagnoses such as myelin oligodendrocyte glycoprotein antibody–associated disease where the host immune system attacks myelin in the brain, optic nerve and spinal cord. The precise mechanisms underlying associations of SARS-CoV-2 infection with a range of demyelinating diseases of the CNS are undefined^11^ but likely to involve triggering of autoimmunity among susceptible hosts through immune-mediated responses to eliminate the virus.^12^ Mechanisms may include the acute infection–related cytokine storm impairing myelination of neurons through an effect on glial cells,^13^ or renin–angiotensin–aldosterone system imbalance may fail to prevent immune system–related damage to myelin^11^ and upregulation of a disintegrin and metalloprotease 17 (ADAM-17) limiting oligodendrocyte maturation and thus myelin formation.^11^

Given the relatively short duration since the beginning of the pandemic and the likely extended duration between infection and multiple sclerosis diagnosis,^6-8^ associations of infection with a first multiple sclerosis diagnosis may be because the infection represents a precipitating event for an already initiated disease process. While initiation of multiple sclerosis pathogenesis cannot be ruled out entirely, it may be more likely that a systemic infection causes an exacerbation^14^ that leads to a multiple sclerosis diagnosis, so it is useful to consider associations of COVID-19 with multiple sclerosis separately from the other demyelinating diagnoses.

We used healthcare and other register data to study the entire population of Sweden aged 3–100 years from January 2020 to identify associations of COVID-19 with subsequent non-multiple sclerosis demyelinating diseases and multiple sclerosis.

## Materials and methods

This register-based study included all individuals aged between 3 and 100 years who were resident in Sweden on 1 January 2020 and after exclusion of those with any demyelinating disease, including multiple sclerosis, (*n* = 21 538), a total of 9 959 818 were included in the analysis. This age range was chosen as the more common demyelinating diseases tend to occur after early infancy in children,^15^ so children under 3 years of age were excluded as there may be greater diagnostic uncertainty in this group. The data for people over 100 years of age are sparse, so the maximum inclusion age was 100 years. The data for the current analysis are part of the Swedish COVID-19 Investigation for Future Insights—a Population Epidemiology Approach using Register Linkage (SCIFI-PEARL) project database, which includes broad sociodemographic and healthcare information of the full general population of Sweden, including all PCR-verified COVID-19 diagnoses.^16^ Most data for the study came from national registers and were available from 1 January 2015 to 30 November 2022 to identify risk factors and temporal trends pre-dating the pandemic. The Total Population Register provided information on dates of birth, death, immigration and emigration, as well as sex, region of residence and region of birth. SmiNet, the national register of notifiable communicable diseases managed by the Public Health Agency of Sweden, was used to identify all individuals with a positive SARS-CoV-2 PCR test. The Patient Register provided information on hospital diagnoses (particularly at the beginning of the pandemic, there may have been a small number of hospital patients with COVID-19 diagnosis not verified by PCR testing). The Patient Register has existed since 1964 for inpatient care, achieving full national coverage in 1987, with the addition of specialist outpatient diagnoses in 2001. The Swedish Intensive Care Register identifies dates of admission to intensive care (either directly or transferred within the hospital), with data available for this study from 1 January 2020.

### Exposures

The marker of more severe SARS-CoV-2 infection was defined as hospital inpatient admission with a diagnosis of COVID-19 (including admission to intensive care, directly or from another inpatient setting) identified using the National Patient Register and Intensive Care registers, using the Swedish version of ICD-10 codes U07.1 or U07.2. Less severe disease was defined as a positive PCR test from SmiNet or an outpatient visit to specialist care with diagnosis codes U07.1 or U07.2 in the National Patient Register, without hospital admission. Where both measures of exposure occurred in the same patient, the marker of more severe disease replaced the less severe in the analysis.

### Outcomes

The outcomes studied were hospital inpatient and outpatient (but not primary care) diagnoses of non-multiple sclerosis demyelinating disease and multiple sclerosis. The non-multiple sclerosis demyelinating diagnoses were defined using ICD-10 codes G36 and G37 (other acute disseminated demyelination and other demyelinating diseases of CNS) from primary or secondary outpatient and inpatient diagnoses recorded in the Patient Register. Multiple sclerosis (G35) was examined separately using primary or secondary outpatient and inpatient diagnoses, as the long asymptomatic/prodromal phases seen in multiple sclerosis ^6-8^ imply that diagnoses shortly after infection may represent an exacerbation of a pre-existing disease processes rather than initiation of pathogenesis.

### Covariates

Data from the Patient Register were used to create a Charlson comorbidity index^17-19^ (categorized as 0, 1, 2 and ≥3) comprising myocardial infarction, congestive heart failure, peripheral vascular disease, cerebrovascular disease, chronic pulmonary disease, rheumatic disease, dementia, liver disease, diabetes mellitus, hemiplegia/paraplegia, renal disease, malignancy, metastatic tumours, peptic ulcer disease and HIV/AIDS. Other variables were sex; year of birth (1920–1940, 1941–1960, 1961–1980, 1981–2000 and 2001–2016); region of birth (Africa, Asia, European Union excluding Nordic countries, Europe excluding European Union and Nordic countries, North America, Nordic countries excluding Sweden, Oceania, former Soviet Union, Sweden, South America and other); and Swedish healthcare region (North, South, Stockholm, South-East, Uppsala-Örebro, West and other).

### Statistical analysis

The characteristics of the participants were summarized using frequencies and percentages, cross-tabulated by highest severity of COVID-19 infection status as defined above by 30 November 2022.

People with a diagnosis of any demyelinating disease, G35, G36, G37 and G040.2 (post-immunization acute disseminated encephalitis, myelitis and encephalomyelitis), prior to 2020 were excluded from the analysis.

The two outcomes were analysed separately: non-multiple sclerosis demyelinating disease and multiple sclerosis. The study population was followed from 1 January 2020 to the first occurrence of the outcome diagnosis, date of emigration, death or 30 November 2022, whichever occurred first. A time-varying variable changed exposure status in the sequence described in the exposures section, with risk estimated from the first occurrence of the exposure indicator. Calendar time was the underlying time scale as design this will help to address timing of virus waves and implementation of policies designed to limit transmission. Stratification was also performed by exposure before or from 1 January 2021, as vaccination against SARS-CoV-2 began towards the end of 2020 in Sweden and the Alpha variant was identified as dominant in early 2021 and then to be replaced by others. We estimated rates of outcomes of interest per 100 000 person-years with 95% confidence intervals (CIs) and used Cox proportional hazards regression to estimate hazard ratios (HRs) with 95% CI and *P*-values. Adjustment was for sex, year of birth (age), Charlson comorbidity index, healthcare region and region of birth. Kaplan–Meier plots were used to present the duration between hospital admission for COVID-19 and diagnosis of non-multiple sclerosis demyelinating disease and multiple sclerosis.

The Schoenfeld residual test assessed the proportional hazards assumption, which was not violated. Statistical significance was defined as CIs that do not include 1.00 and *P* < 0.05.

All analyses were conducted using Stata version 17.0 (StataCorp. LLC).

### Ethical approval

Ethical approval for the SCIFI-PEARL project was obtained from the Swedish Ethical Review Authority (2020-01800 with subsequent amendments).

## Results

Table 1 shows the characteristics of the Swedish population aged 3–100 years in January 2020. The time-varying SARS-CoV-2 infection status variable is tabulated such that individuals were classified to indicate the most serious infection category by the end of follow-up: someone who tested positive and was later admitted to hospital (with COVID-19), only appears in the hospital admission column, even if they have a subsequent positive PCR test without hospital admission. Those who only tested positive without hospital admission tended to be younger adults, while those admitted to hospital with COVID-19 were on average older adults (Table 1). Those admitted to hospital were more likely to have pre-existing poorer health, as indicated by the Charlson comorbidity index. People with any demyelinating disease before baseline were excluded from all subsequent analysis. During follow-up, there were 1139 hospital outpatient or inpatient diagnoses of non-multiple sclerosis demyelinating disease and 2787 of multiple sclerosis.

**Table 1:** Baseline characteristics of the study population (*N* = 9 981 915) aged between 3 and 100 years in January 2020, by subsequent SARS-CoV-2 infection status by 30 November 2022

	No diagnosed infection Total = 7 498 492 *n* (%)	Positive SARS-CoV-2 test only Total = 2 371 402 *n* (%)	Hospital admission due to SARS-CoV-2 Total = 112 021 *n* (%)
Age (years)			
3–10	716 034 (9.6)	205 879 (8.7)	794 (0.7)
11–20	851 939 (11.4)	323 550 (13.6)	1718 (1.5)
21–30	926 137 (12.4)	410 063 (17.3)	5174 (4.6)
31–40	908 194 (12.1)	444 038 (18.7)	7343 (6.6)
41–50	868 125 (11.58)	421 281 (17.8)	10 194 (9.1)
51–60	960 263 (12.8)	317 894 (13.4)	15 874 (14.2)
61–70	958 268 (12.8)	130 613 (5.5)	18 617 (16.6)
71–80	882 771 (11.8)	63 011 (2.7)	26 547 (23.7)
81–90	357 146 (4.8)	41 226 (1.7)	20 809 (18.6)
91–100	69 615 (0.9)	13 847 (0.6)	4951 (4.4)
Sex			
Male	3 856 628 (51.4)	1 100 319 (46.4)	62 231 (55.6)
Female	3 641 864 (48.6)	1 271 083 (53.6)	49 790 (44.5)
Charlson comorbidity index			
0	6 561 231 (87.5)	2 166 234 (91.4)	62 858 (56.1)
1	358 769 (4.8)	101 845 (4.3)	12 966 (11.6)
2	372 423 (5.0)	71 989 (3.0)	16 980 (15.2)
3 or more	206 069 (2.8)	31 334 (1.3)	19 217 (17.2)
Region of Sweden			
North	668 495 (8.9)	180 217 (7.6)	8151 (7.3)
South	1 335 440 (17.8)	436 487 (18.4)	16 198 (14.5)
Stockholm	1 723 729 (23.0)	554 305 (23.4)	31 734 (28.3)
South-East	783 644 (10.5)	230 028 (9.7)	11 224 (10.0)
Uppsala-Örebro	1 500 674 (20.0)	502 513 (21.2)	20 488 (18.3)
West	1 350 693 (18.0)	461 271 (19.5)	17 054 (15.2)
Other	135 817 (1.8)	6 581 (0.3)	7 172 (6.4)
Region of origin			
Africa	180 238 (2.4)	48 191 (2.0)	3 044 (2.7)
Asia	569 864 (7.6)	200 446 (8.5)	12 766 (11.4)
European Union excluding Nordic countries	297 723 (4.0)	77 320 (3.3)	4 408 (3.9)
Europe excluding European Union and Nordic countries	185 408 (2.5)	76 236 (3.2)	5 776 (5.2)
North America	31 692 (0.4)	9 259 (0.4)	393 (0.4)
Nordic countries excluding Sweden	189 343 (2.5)	36 599 (1.5)	4980 (4.5)
Oceania	4 998 (0.1)	1353 (0.1)	19 (0.0)
Former Soviet Union	4 199 (0.1)	1066 (0.0)	121 (0.1)
Sweden	5 981 516 (79.8)	1 899 892 (80.1)	79 093 (70.6)
South America	52 119 (0.7)	20 763 (0.9)	1408 (1.3)
Other	1 392 (0.0)	277 (0.0)	13 (0.0)
Demyelinating disease diagnoses prior to 2020			
No	7 483 154 (99.8)	2 366 117 (99.8)	111 106 (99.2)
Yes	15 338 (0.2)	5285 (0.2)	915 (0.8)

### Non-multiple sclerosis demyelination

**Figure 1:**
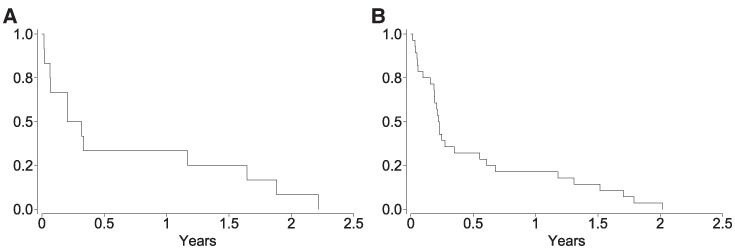
**Timing of demyelinating disease diagnoses following hospital admission for COVID-19**. The Kaplan–Meier curves show the duration from hospital admission for COVID-19, modelled as a time-dependent variable to (**A**) diagnosis of a non-multiple sclerosis demyelinating disease (*n* = 12) and (**B**) diagnosis of multiple sclerosis (*n* = 28).

Hospital admission for SARS-CoV-2 was associated with a statistically significant raised risk of subsequent non-multiple sclerosis demyelinating disease, both before and after adjustment for potential confounding factors (*P* = 0.003), and adjustment did not attenuate the magnitude of association (Table 2). The association with positive test only was attenuated and remained slightly above 1.00 but not statistically significant after adjustment (*P* = 0.541). Females and those with a pre-existing higher Charlson comorbidity index score were also more likely to experience demyelinating disease (Table 2). The median age at diagnosis of demyelination following hospital admission for COVID-19 was 42 years (range 25–89 years), and the median duration between admission for COVID-19 and diagnosis of demyelination was 89 days (range 6–809 days). Figure 1A shows the duration between first hospital admission for COVID-19 and non-multiple sclerosis demyelinating disease diagnoses. The majority of these diagnoses were made in the first 6 months, but one-third of them were diagnosed over a year after admission for COVID-19. The specific demyelination diagnoses associated with admission for COVID-19 are shown in Table 3. There was only a small number of individuals with non-multiple sclerosis demyelinating disease outcomes associated with hospital admission for COVID-19 (*n* = 12). The diseases associated with COVID-19 hospital admission were in the following diagnostic categories: neuromyelitis optica, clinically isolated syndrome and other specified acute disseminated demyelination, central demyelination of the corpus callosum, myelin oligodendrocyte glycoprotein antibody disease and other specified demyelinating diseases of the CNS and unspecified diseases of the CNS.

**Table 2:** HRs with 95% CIs for the association of SARS-CoV-2 status with non-multiple sclerosis demyelinating diseases of the CNS

	Non-multiple sclerosis demyelinating diseases, *n*/total *N*	Rate^c^ (95% CI)	Unadjusted HR (95% CI)	*P*	Adjusted^b^ HR (95% CI)	*P*
Total	1124/9 959 818	4.0 (3.7–4.2)				
SARS-CoV-2^a^						
Not diagnosed	960/9 959 776	3.8 (3.6–4.0)	Reference		Reference	
Positive test only	152/2 386 953	5.1 (4.3– 6.0)	1.30 (1.09–1.56)	0.004	1.06 (0.88–1.27)	0.541
Hospital admission	12/107 282	9.0 (5.1–15.9)	2.33 (1.32–4.12)	0.004	2.36 (1.33–4.19)	0.003
Sex						
Male	386/5 012 336	2.7 (2.4–3.0)	Reference			
Female	738/4 947 482	5.2 (4.9–5.6)	1.94 (1.71–2.19)	<0.001	1.99 (1.76–2.25)	<0.001
Charlson comorbidity index						
0	969/8 772 348	3.8 (3.6–4.1)	Reference		Reference	
1	59/472 485	4.4 (3.4–5.7)	1.14 (0.88–1.49)	0.318	1.50 (1.15–1.95)	0.003
2	71/459 256	5.7 (4.4–7.2)	1.49 (1.17–1.90)	0.001	2.24 (1.75–2.88)	<0.001
3 or more	25/255 729	4.3 (2.9–6.3)	1.12 (0.75–1.67)	0.569	1.88 (1.25–2.83)	0.002

^a^SARS-CoV-2 was modelled as a time-varying exposure; hence, the total sum of number of observations (individuals) contributing time at risk in each category of SARS-CoV-2 exposure is greater than the total number of individuals.

^b^Adjusted for birth year (1920–1940, 1941–1960, 1961–1980, 1981–2000 and 2001–2016), sex (male and female), regions of Sweden (North, South, Stockholm, South-East, Uppsala-Örebro, West and other), region of origin (Africa, Asia, European Union excluding Nordic countries, Europe excluding European Union and Nordic countries, North America, Nordic countries excluding Sweden, Oceania, former Soviet Union, Sweden, South America and other) and Charlson comorbidity index.

^c^Per 100 000 person-years.

**Table 3:** Demyelinating diseases (excluding multiple sclerosis), by SARS-CoV-2 infection status

ICD-10		Not diagnosed, *n* = 960	Positive test only, *n* = 152	Hospital admission, *n* = 12
G36	Other acute disseminated demyelination			
G36.0	Neuromyelitis optica (Devic)	89 (9.3%)	11 (7.2%)	2 (16.7%)
G36.1	Acute and sub-acute haemorrhagic leucoencephalitis (Hurst)		1 (0.7%)	
G36.8	Clinically isolated syndrome and other specified acute disseminated demyelination	9 (0.9%)	4 (2.6%)	1 (8.3%)
G36.9	Acute disseminated demyelination, unspecified	23 (2.4%)	3 (2.0%)	
G37	Other demyelinating diseases of the CNS			
G37.0	Diffuse sclerosis	4 (0.4%)	1 (0.7%)	
G37.1	Central demyelination of corpus callosum	4 (0.4%)		1 (8.3%)
G37.2	Central pontine myelinolysis	38 (4.0%)	1 (0.7%)	
G37.3	Acute transverse myelitis in demyelinating disease of CNS	32 (3.3%)	2 (1.3%)	
G37.4	Sub-acute necrotizing myelitis	2 (0.2%)	1 (0.7%)	
G37.5	Concentric sclerosis (Baló)	1 (0.1%)		
G37.8	Myelin oligodendrocyte glycoprotein antibody disease and other specified demyelinating CNS diseases	67 (7.0%)	10 (6.6%)	1 (8.3%)
G37.9	Demyelinating disease of CNS, unspecified	732 (76.3%)	125 (82.2%)	7 (58.3%)

Some individuals had more than one demyelinating disease diagnosis; thus, column percentages may exceed 100%. The percentages indicate proportions only among those with a demyelinating disease and cannot be used to estimate relative risk.

### Multiple sclerosis

After adjustment for the potential confounding factors, hospital admission for COVID-19 was associated with raised risk of a first multiple sclerosis diagnosis (*P* < 0.001), but only positive test did not represent a raised risk (Table 4). A total of 28 individuals were diagnosed with multiple sclerosis following hospital admission for COVID-19. The association of pre-existing Charlson comorbidity index with multiple sclerosis was inconsistent. The median age at multiple sclerosis diagnosis in those admitted to hospital for infection was 56.7 years (range 15.9–89.9 years). In this group, the median duration between admission for COVID-19 and diagnosis of multiple sclerosis was 82 days (range 5–736 days). Figure 1B shows the duration between first hospital admission for COVID-19 and first multiple sclerosis diagnosis. The majority of these diagnoses were made in the first 6 months, and only approximately one-fifth were diagnosed over a year after admission for COVID-19.

**Table 4:** HRs with 95% CIs for the association of SARS-CoV-2 status with multiple sclerosis

	Multiple sclerosis, *n*/total *N*	Rate^c^ (95% CI)	Unadjusted HR (95% CI)	*P*	Adjusted^b^ HR (95% CI)	*P*
Total	2787/9 959 818	9.8 (9.4–10.2)				
SARS-CoV-2^a^						
Not diagnosed	2403/9 959 776	9.5 (9.1–9.9)	Reference		Reference	
Positive test only	356/2 386 629	11.9 (11.7–13.2)	1.30 (1.16–1.47)	<0.001	1.08 (0.96–1.22)	0.200
Hospital admission	28/107 246	21.1 (14.6–30.5)	2.28 (1.57–3.31)	<0.001	2.48 (1.70–3.61)	<0.001
Sex						
Male	926/5 012 336	6.5 (6.1–6.9)	Reference			
Female	1861/4 947 482	13.2 (12.6–13.8)	2.03 (1.88–2.20)	<0.001	2.08 (1.92–2.25)	<0.001
Charlson comorbidity index						
0	2470/8 772 348	9.8 (9.4–10.2)	Reference		Reference	
1	120/472 485	8.9 (7.5–10.7)	0.91 (0.76–1.10)	0.323	1.14 (0.95–1.37)	0.157
2	149/459 256	12.0 (10.2–14.1)	1.23 (1.04–1.45)	0.015	1.60 (1.35–1.90)	<0.001
3 or more	48/255 729	8.2 (6.2–10.9)	0.84 (0.63–1. 12)	0.227	1.15 (0.86–1.55)	0.342

^a^SARS-CoV-2 was modelled as a time-varying exposure; hence, the total sum of number of observations (individuals) contributing time at risk in each category of SARS-CoV-2 exposure is greater than the total number of individuals.

^b^Adjusted for birth year (1920–1940, 1941–1960, 1961–1980, 1981–2000 and 2001–2016), sex (male and female), regions of Sweden (North, South, Stockholm, South-East, Uppsala-Örebro, West and other), region of origin (Africa, Asia, European Union excluding Nordic countries, Europe excluding European Union and Nordic countries, North America, Nordic countries excluding Sweden, Oceania, former Soviet Union, Sweden, South America and other) and Charlson comorbidity index.

^c^Per 100 000 person-years.

### Infection before 1 January 2021 and subsequently

When exposure was separated into before and from 1 January 2021, there was no notable difference for non-multiple sclerosis demyelination, but a non-conclusive suggestion of somewhat higher magnitude multiple sclerosis risk was associated with hospital admission for COVID-19 after 2020 (Table 5).

**Table 5:** HRs with 95% CIs for the association of SARS-CoV-2 status, before and from 1 January 2021, with non-multiple sclerosis demyelinating disease of the CNS and multiple sclerosis

	Non-multiple sclerosis demyelinating diseases, *n*/total *N*	Adjusted^b^ HR (95% CI)	*P*	Multiple sclerosis, *n*/total *N*	Adjusted^b^ HR (95% CI)	*P*
Total	1124/9 959 818			2787/9 959 818		
SARS-CoV-2^a^						
Not diagnosed	960/9 959 776	Reference		2403/9 959 776	Reference	
Positive test only before 1 January 2021	42/438 017	0.96 (0.71–1.32)	0.823	112/437 987	1.10 (0.91–1.34)	0.319
Positive test only from 1 January 2021	110/1 948 936	1.10 (0.89–1.36)	0.366	244/1 948 642	1.07 (0.93–1.23)	0.332
Hospital admission before 1 January 2021	7/39 576	2.60 (1.23–5.49)	0.012	11/39 564	1.85 (1.02–3.36)	0.042
Hospital admission from 1 January 2021	5/67 706	2.09 (0.86–5.05)	0.102	17/67 682	3.18 (1.97–5.13)	<0.001

^a^SARS-CoV-2 was modelled as a time-varying exposure; hence, the total sum of number of observations (individuals) contributing time at risk in each category of SARS-CoV-2 exposure is greater than the total number of individuals.

^b^Adjusted for birth year (1920–1940, 1941–1960, 1961–1980, 1981–2000 and 2001–2016), sex (male and female), regions of Sweden (North, South, Stockholm, South-East, Uppsala-Örebro, West and other), region of origin (Africa, Asia, European Union excluding Nordic countries, Europe excluding European Union and Nordic countries, North America, Nordic countries excluding Sweden, Oceania, former Soviet Union, Sweden, South America and other) and Charlson comorbidity index.

## Discussion

This national study of SARS-CoV-2 infection found that hospital admission for COVID-19 was associated with an increased risk of non-multiple sclerosis demyelinating diseases and an increased risk of a multiple sclerosis diagnosis.

While this study provides some of the most comprehensive evidence to date that SARS-CoV-2 infection is associated with subsequent demyelinating diseases of the CNS, it should be considered that the association might be explained by shared susceptibility or surveillance bias (medical management of one condition results in diagnosis of another). Earlier research has suggested this association but was based only on small studies with methodological limitations, as reported in a systematic review that summarized evidence of possible associations with encephalomyelitis, brain demyelination, transverse myelitis, neuromyelitis optica and MOG antibody–associated disease.^1^ The review further identified a total of three individuals with multiple sclerosis–like demyelination, with symptomatic infection pre-dating onset of neurological symptoms by several weeks in the previous studies.^1,20,21^ However, one of these previous studies found that the characteristics of the demyelination were not typical for multiple sclerosis, suggesting post-viral demyelination rather than true multiple sclerosis.^1^ Another systematic review concluded that CNS demyelinating events associated with SARS-CoV-2 infections occurred at a low rate, consistent with the low rates observed in our study.^2^ The current study benefits from follow-up of a national population, with prospectively recorded exposures and greater power to detect rare outcome events.

Our findings are consistent with other research showing that a variety of infections, with likely direct or indirect access to the CNS, are associated with an increased risk of multiple sclerosis.^8,22^ The first focus of this study was on demyelination excluding multiple sclerosis, due to the likely long asymptomatic and prodromal periods of multiple sclerosis,^6-8^ as there may be insufficient time between infection and frank symptomatic onset of multiple sclerosis in most people who may develop the disease in this population. Despite this, there was a raised risk of new multiple sclerosis diagnoses associated with hospital admission for SARS-CoV-2 infection. The numbers are small, so this may represent a minority of patients with more rapid-onset initiation of multiple sclerosis pathogenesis. An alternative explanation is that the sub-clinical multiple sclerosis disease process had already begun but was previously undiagnosed in these individuals. The infection may have resulted in more rapid disease progression such that multiple sclerosis disease activity was no longer sub-clinical, leading to a diagnosis: both bacterial and viral infections are linked with multiple sclerosis exacerbations and disease progression.^23^ The majority of diagnoses of both outcomes occurred during the first 6 months after hospital admission for COVID-19, which could indicate rapid onset of demyelinating disease, precipitation of a pre-existing disease process or diagnoses made due to surveillance bias resulting from increased contact with healthcare professionals. A minority of the demyelinating disease diagnoses were made over a year after hospital admission for COVID-19, which not only eliminates the possibility of surveillance bias due to continued increased healthcare contact but also suggests the importance of longer term follow-up to identify associations with delayed disease onset.

There is evidence from this study of associations of SARS-CoV-2 infection with CNS demyelination (the extent to which this is a causal association requires more evidence), but there is a question over whether the pandemic will have an influence on future risk beyond what is reported here. There is typically a 10–20-year duration between exposure to an environmental risks factor, including acute infections^6-8^ and other exposures,^24,25^ and a multiple sclerosis diagnosis. It should however be noted that the vast majority of those who go on to develop multiple sclerosis do not have a diagnosed demyelinating event or other evidence of early multiple sclerosis onset in the years immediately following infections or other environmental exposures linked with multiple sclerosis risk.^6,8,25^ Given the possible CNS involvement in SARS-CoV-2 infection,^26^ there may be a possibility of future demyelinating disease diagnoses, but perhaps this will be largely among those with more severe COVID-19, as shown previously for other infections with direct or indirect access to the CNS.^6,8,22^

Research using animal models has indicated that corona virus infections can give rise to immune dysregulation, resulting in multiple sclerosis–like demyelination,^27^ and are associated with upregulation of a range of pro-inflammatory cytokines that can cross the blood–brain barrier, creating a highly pro-inflammatory environment in the CNS.^28^ More specifically, increased levels of IL-17 have been observed in coronavirus–infected patients,^29^ which has been implicated in multiple sclerosis aetiology based on animal models.^30^ It is also possible that some respiratory infections result in autoreactive T-cells in the lungs that enter bronchus-associated lymphoid tissue and can then cross the blood–brain barrier, resulting in CNS inflammation.^31^ Respiratory infections, likely including COVID-19, can induce a local and systemic T-helper (Th)17 response, including memory Th17 cells, and mechanisms such as molecular mimicry could result in Th17 reactivation and CNS infiltration.^32^ Thus in general, lung and bronchus-associated lymphoid tissue have been implicated in pathogenesis of autoimmune neuro-inflammation^31^ and thus indicating the potential plausibility of COVID-19 influencing demyelination risk.

While this study is both large and comprehensive and identified all known diagnoses of COVID-19 in Sweden during the follow-up period, there are also some potential limitations. An important limitation is that the only measure of greater COVID-19 severity that we could use was whether the infection resulted in hospital admission. This does not identify specific symptoms of potential importance or other factors that may influence the decision to admit someone with the infection, so there is likely to be some heterogeneity in this putative marker of severity. The number of individuals with the outcome diagnoses of non-multiple sclerosis demyelinating diseases is small, and more than half of them have a non-specific diagnosis, which may question diagnostic accuracy so the results should be interpreted with caution. The Swedish ICD codes for the outcome diagnoses were limited to one digit after the decimal point, so some specific diagnoses were grouped. While this was not a problem for identification of multiple sclerosis, other groups contained diagnoses, which would have been interesting to identify separately, such as clinically isolated syndrome, which is associated with future multiple sclerosis risk,^33^ and myelin oligodendrocyte glycoprotein antibody disease, which is a separate disease entity.^34^ Lack of a specific diagnosis may also be because of insufficient follow-up time. However, in the context of all demyelinating diagnoses, multiple sclerosis is less rare. As diagnostic precision remains an issue, and disease progression is incompletely described, further research should examine evidence of progression of demyelination with information on diagnostic methods, including use of repeated MRI examinations. Many further individuals may have been infected but were not tested by PCR (however, testing during the major period of the study period was extensive, although there was limited testing at the beginning of the pandemic). As we divided the analysis into two periods of infection, this may have helped to identify changes in the PCR-positive population, particularly those who were not admitted to hospital. Some demyelinating diseases, particularly multiple sclerosis, are insidious in onset, with development over a long period, and so will mostly not have been detected during the limited follow-up time of this study. The SARS-CoV-2 viral variant responsible for the infection could not be identified in our data, so calendar period was used as the underlying timescale in the analysis to take aspects of the changing characteristics of the pandemic and vaccine coverage into account. We also divided the period of exposure, up to the end of 2020 and then subsequently. Shared susceptibility to SARS-CoV-2 infection and the outcomes could explain some of the associations. To address this, anyone with a demyelinating diagnosis prior to 2020 was excluded, and we adjusted for Charlson comorbidity index score to control for underlying health problems: as expected, a higher Charlson comorbidity index score was generally positively associated with the outcomes, but the demyelinating outcomes associated with COVID-19 were found most notably among those without comorbidity. Another limitation is that we did not have information on body mass index, which would have been a potentially useful covariate as obesity is an important risk factor for severe COVID-19. Importantly, the results may have been influenced by surveillance/referral bias, as continued contact with healthcare following hospital admission for COVID-19 may have resulted in diagnosis of demyelinating disease.

We found an increased risk of demyelinating diseases of the CNS among people treated in hospital for COVID-19. A proportion of these associations with COVID-19 may be due to shared susceptibility or surveillance bias. The occurrence of delayed-onset demyelinating diseases should continue to be assessed among those who experienced COVID-19, as some of these diseases can have long asymptomatic and prodromal phases.

## Supplementary Material

fcae406_Supplementary_Data

## Data Availability

The data used in this study are de-identified individual-level data from Swedish healthcare registers and can be obtained from the respective Swedish public data holders with ethical approval for the research in question, subject to relevant legislation, processes and data protection. Details of the data can be found at: https://www.gu.se/en/research/scifi-pearl. The codes generated and used in this work can be found in the Supplementary material and are available online at *Brain Communications*.
