# Supplementary material for: SARS-CoV-2 infection and risk of subsequent demyelinating diseases: national register–based cohort study
Source: Brain Commun. 2024 Nov 29;6(6):fcae406. doi: 10.1093/braincomms/fcae406 (PMC11629974; doi:10.1093/braincomms/fcae406)
Supplement: fcae406_Supplementary_Data [file fcae406_supplementary_data.zip › Original_submission_manuscript.pdf]

OXFORD UNIVERSITY PRESS | Brain Communications

SARS-CoV-2 infection and risk of subsequent demyelinating diseases: national register-based cohort study

Journal:	*Brain Communications*
Manuscript ID	BRAINCOM-2024-038
Manuscript Type:	Original Article
Date Submitted by the Author:	19-Jan-2024
Complete List of Authors:	Montgomery, Scott; Örebro University; Karolinska Institutet, Department of Medicine; University College London, Department of Epidemiology and Public Health<br>Vingeliene, Snieguole; Örebro University<br>Li, Huiqi; University of Gothenburg<br>Backman, Helena; Örebro University, Department of Obstetrics and Gynaecology<br>Udumyan, Ruzan; Örebro University<br>Jendeberg, Johan; Örebro University, Department of Radiology<br>Rasmussen, Gunlög; Örebro University, Department of Infectious Diseases<br>Sundqvist, Martin; Örebro University, Department of Laboratory Medicine Clinical Microbiology<br>Fall, Katja; Örebro University; Karolinska Institutet<br>Hiyoshi, Ayako; Örebro University<br>Nyberg, Fredrik; University of Gothenburg, School of Public Health and Community Medicine, Institute of Medicine, Sahlgrenska Academy
Keywords:	SARS-CoV-2, multiple sclerosis, demyelinating disease

SCHOLARONE™ Manuscripts

SARS-CoV-2 infection and risk of subsequent demyelinating diseases: national register-based cohort study

Scott Montgomery, professor, scott.montgomery@oru.se (0000-0001-6328-5494),[1 2 3]
Snieguole Vingeliene, epidemiologist, snieguole.vingeliene@oru.se,[1]
Huiqi Li, associate professor, huiqi.li@gu.se,[4]
Helena Backman, associate professor, Helena.Backman@oru.se (0000-0002-2691-7525)[5]
Ruzan Udumyan, epidemiologist, ruzan.udumyan@regionorebrolan.se,[1]
Johan Jendeberg, radiologist, johan.jendeberg@regionorebrolan.se (0000-0001-8949-119X)[6]
Gunlög Rasmussen, senior consultant, gunlog.rasmussen@regionorebrolan.se,[7]
Martin Sundqvist, senior consultant, Martin.Sundqvist@regionorebrolan.se,[8]
Katja Fall, professor, Katja.Fall@oru.se,[1 9]
Ayako Hiyoshi, associate professor, ayako.hiyoshi@oru.se (0000-0002-2088-0530),[1]
Fredrik Nyberg, professor, fredrik.nyberg.2@gu.se (0000-0003-0892-5668)[4]

1. Clinical Epidemiology and Biostatistics, School of Medical Sciences, Faculty of Medicine and Health, Örebro University, Örebro, Sweden.
2. Clinical Epidemiology Division, Department of Medicine, Solna, Karolinska Institutet, Stockholm, Sweden.
3. Department of Epidemiology and Public Health, University College London, London, UK.
4. School of Public Health and Community Medicine, Institute of Medicine, Sahlgrenska Academy, University of Gothenburg, Gothenburg, Sweden.
5. Department of Obstetrics and Gynaecology, Faculty of Medicine and Health, Örebro University, Örebro, Sweden.
6. Department of Radiology, Faculty of Medicine and Health, Örebro University, Örebro, Sweden.
7. Department of Infectious Diseases, School of Medical Sciences, Faculty of Medicine and Health, Örebro University, Örebro, Sweden.
8. Department of Laboratory Medicine, Clinical Microbiology, Faculty of Medicine and Health, Örebro University, Örebro, Sweden.
9. The Institute of Environmental Medicine, Karolinska Institutet, Stockholm, Sweden

Correspondence to
Prof. Scott Montgomery
Clinical Epidemiology and Biostatistics
School of Medical Sciences
Faculty of Medicine and Health
Örebro University
Campus USÖ
Södra Grev Rosengatan 30
703 62 Örebro
Sweden

Telephone +46 727236210

For Review Only

Abstract

Multiple sclerosis risk is associated with prior infectious exposures, so we assessed whether SARS-CoV-2 infection is associated with subsequent diagnoses of non-multiple sclerosis demyelinating diseases, multiple sclerosis, and infectious mononucleosis due to Epstein-Barr virus, which is an important MS risk factor. All residents of Sweden aged 3 to 100 years were followed between 1st January 2020 and 30th November 2022, excluding those with demyelinating disease prior to 2020, comprising 9,981,915 individuals divided into uninfected, and those who were infected were categorised into those with and without hospital admission for the infection as a marker of infection severity. Cox regression assessed the risk of three separate outcomes: hospital diagnosed non-multiple sclerosis demyelinating diseases of the central nervous system, multiple sclerosis, and infectious mononucleosis due to Epstein-Barr virus. The exposures were modelled as time-varying covariates (uninfected, infection without hospital admission and infected with hospital admission). Hospital admission for COVID-19, was associated with raised risk of subsequent non-multiple sclerosis demyelinating disease. Rates per 100 000 person years (and 95% confidence intervals) were 3.8 (3.6 to 4.1) among those without a COVID-19 diagnosis and 9.0 (5.1 to 15.9) among those admitted to hospital for COVID-19, with an adjusted hazard ratio and (and 95% confidence interval) of 2.31 (1.30 to 4.10). Equivalent associations with multiple sclerosis were rates of 9.5 (9.1 to 9.9) and 21.0 (14.5 to 30.5), and an adjusted hazard ratio of 2.48 (1.70 to 3.61). For subsequent infectious mononucleosis caused by Epstein-Barr virus, hospital admission for COVID-19 was associated with a rate of 10.5 (6.2 to 17.8) compared with 4.7 (4.4 to 5.0) for those without COVID-19, and an adjusted hazard ratio of 5.63 (3.29 to 9.66). Hospital admission for COVID-19 was associated with a subsequent raised risk of demyelinating diseases of the central nervous system. COVID-19 was also associated with a raised risk of infectious mononucleosis caused by Epstein-Barr virus, an established risk

For Review Only

factor for MS. Greater duration of follow-up will assist further in differentiating between causal associations and potentially shared susceptibility or surveillance bias for the associations of COVID-19 with risk of multiple sclerosis and other demyelinating diseases, which can have long asymptomatic and prodromal phases.

Keywords: SARS-CoV-2, multiple sclerosis, demyelinating disease

For Review Only

Introduction

Viral infections, particularly due to Epstein-Barr virus (EBV), have been linked with increased risk of subsequent multiple sclerosis (MS).[1-3] Given the evidence that SARS-CoV-2 infection can have consequences for the central nervous system (CNS), including autoimmune disorders,[4] how likely is it that it increases risk of MS and other demyelinating diseases of the CNS? There is an extended asymptomatic/prodromal period in MS, and there may even be a 10-to-20-year duration between an acute triggering infection and an MS diagnosis.[3 5 6] It may therefore be premature to assess fully the extent to which the pandemic may result in increased numbers of patients with MS, but early indicators of risk may already be detectable, including non-MS demyelinating diagnoses and EBV infectious mononucleosis.

As MS develops there can be earlier demyelinating events, signalling pathogenesis. For example, optic neuritis can be an early sign of MS.[7] Even though many MS patients will not have had a clinically identified demyelinating event in the years immediately following an infection linked with MS risk, associations of SARS-CoV-2 infections with demyelination will signal the possibility that the infection also increases MS risk. A systematic review of CNS demyelination following SARS-CoV-2 infection concluded that there are some preliminary but inconclusive data on an association, but with limitations, as it was based on a small number of case reports and case series.[8]

Given the relatively short duration since the beginning of the pandemic and the likely extended duration between infection and MS diagnosis,[3 5 6] associations of infection with a first MS diagnosis may be because the infection represents a precipitating event for an already initiated disease process. While initiation of MS pathogenesis cannot be ruled-out, it may be more likely that a systemic infection causes an exacerbation[9] that leads to an MS diagnosis, so it is useful

For Review Only

to consider associations of COVID-19 with MS separately from the other demyelinating diagnoses.

While COVID-19 may trigger demyelination and increase future MS risk,[8] this risk may be magnified if the infections result in greater accumulation of further MS risk factors. Infection by SARS-CoV-2 has been shown to result in long-term immune system perturbation,[10] and increased risk of viral reactivation or opportunistic infection, including EBV. Infectious mononucleosis (IM) due to EBV is an important risk factor for MS.[1-3]

We used healthcare and other register data to study the entire population of Sweden aged three to 100 years from January 2020 to identify associations of COVID-19 with subsequent non-MS demyelinating diseases, MS and IM due to EBV.

Materials and methods

This register-based study included all individuals aged between three and 100 years who were resident in Sweden on 1st of January 2020 (N=9 981 915). This age range was chosen as the more common demyelinating diseases tend to occur after early infancy in children,[11] so children under age three years were excluded as there may be greater diagnostic uncertainty in this group. The data for people over age 100 years are sparse, so the maximum inclusion age was 100 years. The data for the current analysis are part of the SCIFI-PEARL (Swedish COVID-19 Investigation for Future Insights - a Population Epidemiology Approach using Register Linkage) project database, which includes broad sociodemographic and healthcare information on the full general population of Sweden, including all PCR-verified COVID-19 diagnoses.[12] Most data for the study were available from 1st of January 2015 to 30th November 2022. The Total Population Register provided information on dates of birth, death, immigration, and

For Review Only

emigration, as well as sex, region of residence and country of birth. SmiNet, the national register of notifiable communicable diseases managed by the Public Health Agency of Sweden, was used to identify all individuals with a positive SARS-CoV-2 PCR test. The Patient Register provided information on hospital diagnoses (particularly at the beginning of the pandemic, there may have been a small number of hospital patients with COVID-19 diagnosis not verified by PCR testing). The Patient Register has existed since 1964 for inpatient care, achieving full national coverage in 1987, with the addition of specialist outpatient diagnoses in 2001. The Swedish Intensive Care Register identifies dates of admission to intensive care (either directly or transferred within the hospital), with data available for this study from 1st January 2020.

Exposures

The marker of more severe SARS-CoV-2 infection was defined as hospital inpatient admission with a diagnosis of COVID-19 (including admission to intensive care, directly or from another inpatient setting) identified using the National Patient Register (NPR) and Intensive Care registers, using the Swedish version of International Classification of Diseases 10 (ICD-10) codes U07.1 or U07.2. Less severe disease was defined as a positive PCR test from SmiNet or an outpatient visit to specialist care with diagnosis codes U07.1 or U07.2 in the NPR, without hospital admission. Where both measures of exposure occurred in the same patient, the marker of more severe disease replaced the less severe in the analysis.

Outcomes

The three outcomes studied were hospital outpatient and inpatient diagnoses of non-MS demyelinating disease, MS, and infectious mononucleosis caused by EBV. The non-MS demyelinating diagnoses were defined using ICD-10 codes G36 and G37 (other acute disseminated demyelination and other demyelinating diseases of central nervous system) from

For Review Only

primary or secondary outpatient and inpatient diagnoses recorded in the Patient Register. MS (G35) was examined separately using primary or secondary outpatient and inpatient diagnoses, as the long asymptomatic/prodromal phases seen in MS[3 5 6] imply that diagnoses shortly after infection may represent an exacerbation of a pre-existing disease processes rather than initiation of pathogenesis. Infectious mononucleosis caused by EBV is an important risk for subsequent MS,[3 6] and this was identified as a primary or secondary outpatient or inpatient hospital diagnosis in the Patient Register using ICD-10 code B27.0.

Covariates

Data from the Patient Register were used to create a Charlson Comorbidity Index[13-15] (categorized as 0, 1, 2, ≥3) comprising myocardial infarction, congestive heart failure, peripheral vascular disease, cerebrovascular disease, chronic pulmonary disease, rheumatic disease, dementia, liver disease, diabetes mellitus, hemiplegia/paraplegia, renal disease, malignancy, metastatic tumours, peptic ulcer disease, and HIV/AIDS. Other variables were sex; year of birth (1920-1940, 1941-1960, 1961-1980, 1981-2000, 2001-2016); country of birth (Africa, Asia, European Union excluding Nordic countries, Europe excluding European Union and Nordic countries, North America, Nordic countries excluding Sweden, Oceania, former Soviet Union, Sweden, South America, and other); and Swedish healthcare region (North, South, Stockholm, South-East, Uppsala-Örebro, West, and other).

Statistical analysis

The characteristics of the participants were summarized using frequencies and percentages, cross-tabulated by highest severity of COVID-19 infection status as defined above by 30th November 2022. Additional analysis examined outcomes associated with admission to intensive care with COVID-19, compared with the no diagnosis category.

For Review Only

People with a diagnosis of any demyelinating disease, G35, G36, G37 and G040.2 (post-immunization acute disseminated encephalitis, myelitis and encephalomyelitis) prior to 2020 were excluded from the analysis.

The three outcomes were analysed separately: non-MS demyelinating disease, MS, and infectious mononucleosis due to EBV. The study population was followed from 1st January 2020 to the first occurrence of the outcome diagnosis, date of emigration, death, or 30th November 2022, whichever occurred first. A time-varying variable changed exposure status in the sequence described in the exposures section, with risk estimated from the first occurrence of the exposure indicator. Calendar time was the underlying time scale as by design this will address timing of virus waves and implementation of policies designed to limit transmission. The results were also stratified by sex, Charlson comorbidity index, age, and country of origin. Stratification was also performed by exposure before or from 1st January 2021, as vaccination against SARS-CoV-2 began towards the end of 2020 in Sweden and the Alpha variant was identified as dominant in early 2021, then to be replaced by others. We estimated rates of outcomes of interest per 100 000 person-years with 95% confidence intervals (CI) and used Cox proportional hazards regression to estimate hazard ratios with 95% CI. Adjustment was for sex, year of birth (age), Charlson Comorbidity Index, healthcare region and country of birth.

The Schoenfeld residual test assessed the proportional hazards assumption, which was not violated. Statistical significance was defined as confidence intervals that do not include 1.00.

All analyses were conducted using Stata version 17.0 (StataCorp LLC).

For Review Only

Ethical Approval

Ethical approval for the SCIFI-PEARL project was obtained from the Swedish Ethical Review Authority (2020-01800 with subsequent amendments).

Results

Table 1 shows the characteristics of the Swedish population aged three to 100 years in January 2020. The time-varying SARS-CoV-2 infection status variable is tabulated such that individuals were classified to indicate the most serious infection category by the end of follow-up: someone who tested positive and was later admitted to hospital (with COVID-19, only appears in the hospital admission column, even if they have a subsequent positive PCR test without hospital admission. Those who only tested positive without hospital admission tended to be younger adults, while those admitted to hospital with COVID-19 were on average older adults (Table 1). Those admitted to hospital were more likely to have pre-existing poorer health, as indicated by the Charlson Comorbidity Index and demyelinating disease prior to 2020. People with any demyelinating disease before baseline were excluded from all subsequent analysis. During follow-up there were 1139 hospital outpatient or inpatient diagnoses of non-MS demyelinating disease, 2787 of MS and 1439 of IM.

Non-MS demyelination

Hospital admission for SARS-CoV-2 was associated with a statistically significant raised risk of subsequent non-MS demyelinating disease, both before and after adjustment for potential confounding factors, and adjustment did not attenuate the magnitude of association (table 2). The association with positive test only was attenuated and remained slightly above 1.00 but not statistically significant after adjustment. Females and those with a pre-existing higher Charlson Comorbidity Index score were also more likely to experience demyelinating disease (table 2).

For Review Only

The median age at diagnosis of demyelination following hospital admission for COVID-19 was 42 years (range, 25 to 89 years) and median duration between admission for COVID-19 and diagnosis of demyelination was 89 days (range, six to 809 days). The specific demyelination diagnoses associated with admission for COVID-19 are shown in Table 3. The outcomes were rare, but those with a raised risk associated with COVID-19 hospital admission were neuromyelitis optica, other specified acute disseminated demyelination, central demyelination of the corpus callosum, other specified demyelinating diseases of the CNS and unspecified diseases of the central nervous system.

MS

After adjustment for the potential confounding factors, hospital admission for COVID-19 was associated with raised risk of a first MS diagnosis (table 4), but positive test only did not represent a raised risk. The association of pre-existing Charlson Comorbidity Index with MS was inconsistent. The median age at MS diagnosis in those admitted to hospital for infection was 56.7 years (range, 15.9 to 89.9 years). In this group, the median duration between admission for COVID-19 and diagnosis of MS was 82 days (range, 5 to 736 days).

IM due to EBV

A positive test only without hospital admission for COVID-19 and hospital admission for COVID-19 were both associated with a raised risk of subsequent hospital diagnosis of IM due to EBV, which is an important risk factor for MS (Table 5). Females were at somewhat greater risk of IM. There were statistically significant associations with higher Charlson Comorbidity Index scores. The median duration between SARS-CoV-2 infection and IM was 228.5 days for those not admitted to hospital but test-positive for COVID-19, and 51.5 days for those admitted to hospital with COVID-19.

For Review Only

Among people who were admitted to hospital for COVID-19 and who also had a diagnosis of non-MS demyelinating disease or MS, there was no record of IM diagnoses between the dates of SARS-CoV-2 infection and diagnosis of demyelinating disease.

Stratified analysis

Stratified analysis found no notable difference by sex for associations with any of the outcomes (Table 6). Associations with non-MS demyelinating disease and MS were more often found among those without comorbidity, while although this was true for the majority with COVID-19 and IM, there was also a proportion with comorbidity. COVID-19 between 11 and 50 years was most notably associated with non-MS demyelination, while infection from age 11 years was most commonly associated with MS. COVID-19 across the age categories was associated with EBV-IM (although the estimate for risk among those with COVID-19 under age 11 years was based on the outcome in only one individual, so was measured imprecisely with wide confidence intervals). When exposure was separated into before and from 1st January 2021, the association with non-MS demyelination was of somewhat higher magnitude prior to 2021, although associations with MS and EBV-IM were of higher magnitude for COVID-19 from 2021 (Table 7).

For Review Only

Discussion

This national study of SARS-CoV-2 infection found that hospital admission for COVID-19 was associated with an increased risk of non-MS demyelinating diseases, as well as an increased risk of an MS diagnosis. The infection was also associated with a raised risk of hospital-diagnosed EBV-associated infectious mononucleosis (IM), which is an important risk factor for MS.[3]

While this study provides some of the most comprehensive evidence to date that SARS-CoV-2 infection is associated with subsequent demyelinating diseases of the CNS, it should be considered that at least some of the association might be explained by shared susceptibility or surveillance bias. Earlier research has suggested this association but was based only on small studies with methodological limitations, as reported in a systematic review that summarized evidence of possible associations with encephalomyelitis, brain demyelination, transverse myelitis, neuromyelitis optica and MOG antibody-associated disease.[8] The review further identified a total of three individuals with MS-like demyelination, with symptomatic infection pre-dating onset of neurological symptoms by several weeks in the previous studies.[8 16 17] However, one of these previous studies found the characteristics of the demyelination was not typical for MS, suggesting post-viral demyelination rather than true MS.[8] The current study benefits from follow-up of a national population, with greater power to detect prospectively recorded exposures and rare outcome events.

Our findings are consistent with other research showing that a variety of infections, with likely direct or indirect access to the CNS, are associated with an increased risk of MS.[6 18] The first focus of this study was on demyelination excluding MS, due to the likely long asymptomatic and prodromal periods of MS,[3 5 6] as there may be insufficient time between infection and frank symptomatic onset of MS in most people who may develop the disease in this population. Despite this, there was a raised risk of new MS diagnoses associated with hospital admission for SARS-CoV-2 infection. The numbers are small, so this may represent a minority of patients with more rapid-onset initiation of MS pathogenesis. An alternative explanation is that the MS disease process had already begun but was undiagnosed in these individuals. The infection may have resulted in an exacerbation and disease progression, such that symptomatic onset led to an

For Review Only

MS diagnosis: both bacterial and viral infections are linked with MS exacerbations and disease progression.[19]

In addition to identifying associations with CNS demyelination, some of which may presage MS, and MS itself, we also examined whether one of the most notable environmental risk factor for MS, EBV-associated IM,[1-3] was more common after SARS-CoV-2 infection, even though policy responses to the pandemic in some settings resulting in social isolation may have reduced overall EBV transmission.[20] Unlike the other outcomes, there was an increased risk of IM not only for those admitted to hospital for COVID-19, but also after COVID-19 without hospital admission. This may be because of immune system perturbation[10] resulting in a more intense immune response against EBV resulting in hospital admission: hospital treated IM is associated with greater MS risk.[3 6] COVID-19 has also been associated with a raised risk of opportunistic infections.[21] However, the results for IM should be interpreted with some caution, as it is possible that people with a recent history of COVID-19 may be referred more readily for hospital treatment for IM as a precautionary measure against new symptoms of SARS-CoV-2 infection and IM treated in primary care (where it is most commonly diagnosed) will not be identified in our study, although more severe acute disease should be captured. One possibility to explain our findings of raised risks for demyelinating disease is that as COVID-19 is associated with increased risk of IM, and in turn it is the EBV-related infection that increases the risk of subsequent demyelinating disease. This seems unlikely to explain the association of COVID-19 with demyelinating disease here, as there were no diagnoses of IM between dates of hospital admission for COVID-19 and diagnosis of non-MS demyelinating disease or MS among those who experienced both exposure and outcomes.

It should be noted that multiple stratified analyses, particularly when outcomes are somewhat uncommon (particularly for non-MS demyelination), may produce chance results that should

For Review Only

be interpreted with caution. There were no notable differences in relative risk of the outcomes associated with COVID-19 when stratified by sex (even though more females than males develop MS). No non-MS demyelinating diseases, nor MS, associated with COVID-19 were diagnosed before age 11 years: non-MS demyelination was diagnosed in earlier adulthood than MS, consistent with non-MS demyelination as a risk factor for subsequent MS. EBV-IM associated with prior COVID-19 occurred at all ages, but was very rare before age 11 years, which is consistent with more severe acute IM resulting in hospital admission when it occurs in adolescence and subsequently. EBV-IM associated with previous COVID-19 was observed throughout adulthood, indicating this outcome was not only present during adolescence when IM is most common. The associations of hospital admission for COVID-19 with the outcomes were most notable in those without any Charlson comorbidity diagnoses, thus reducing, but not eliminating, the possibility that the outcomes are more often detected because of surveillance bias as hospital admission for one disease increases the possibility of an incidental diagnosis of another. As the outcome diagnoses were on average made some time subsequently to hospital admission for COVID-19 this reduces, but does not eliminate, the possibility of surveillance bias accounting for a proportion of the associations. Stratification by exposure before or after 1st January 2021 was performed as vaccination against SARS-CoV-2 began at the end of 2020 in Sweden and the Alpha variant was identified as dominant in early 2021, then to be replaced by others. Both MS and IM were more notably associated with COVID-19 during the latter period, while non-MS demyelination was somewhat more notably associated with infection during the earlier period.

There is evidence from this study of associations of SARS-CoV-2 infection with CNS demyelination and EBV-associated IM, but there is a question over whether the pandemic will have an influence on MS risk beyond what is reported here. There is typically a 10-to-20-year

For Review Only

duration between exposure to an environmental risks factor, including acute infections[3 5 6] and other exposures,[22 23] and an MS diagnosis. Therefore, it is plausible that any notable change in MS incidence due to the pandemic may not be observed for some years. The association with CNS demyelination is consistent with a raised risk for MS, but the absolute number of people with these diagnoses is small. It should however be noted that the vast majority of those who go on to develop MS do not have a diagnosed demyelinating event or other evidence of early MS-onset in the years immediately following infections or other environmental exposures linked with MS risk.[3 6 23] Given the possible CNS involvement in many with SARS-CoV-2 infection,[24] an increase in MS incidence is plausible, but mostly only among those with more severe COVID-19, as shown previously for other infections with direct or indirect access to the CNS[6] requiring hospital treatment[3 6 18]

Research using animal models has indicated that corona-virus infections can give rise to immune dysregulation, resulting in MS-like demyelination[25] and are associated with upregulation of a range of proinflammatory cytokines that can cross the blood-brain barrier, creating a highly proinflammatory environment in the CNS.[26] More specifically, increased levels of IL-17 have been observed in coronavirus infected patients[27], which has been implicated in MS aetiology based on animal models.[28] It is also possible that some respiratory infections result in autoreactive T cells in the lungs that enter bronchus-associated lymphoid tissue (BALT) and can then cross the blood-brain barrier, resulting in CNS inflammation.[29] Respiratory infections, likely including COVID-19, can induce a local and systemic T-helper (Th)17 response, including memory Th17 cells, and mechanisms such as molecular mimicry could result in Th17 reactivation and CNS infiltration.[30] Thus, in general lung and BALT have been implicated in pathogenesis of autoimmune neuro-inflammation,[29] and thus further supporting the plausibility of COVID-19 influencing demyelination risk. The mechanisms

For Review Only

linking SARS-CoV-2 with demyelinating disease may share similarities with the mechanisms linking EBV with MS risk. For example, EBV proteins such as BRRF2, EBNA-1, and the small capsid protein, BFRF3, share epitopes with myelin.[31 32]Antibodies to these proteins can cross-react with myelin antigens leading to autoimmune damage to the CNS.[31 32]Another possible mechanism involves EBV infecting resting B cells, producing immortalized lymphoblastoid cell lines, and causing production of EBNA-1 through EBNA-6 and latent membrane proteins (LMP-1, LMP-2A, and LMP-2B).[33] EBV viraemia following reactivation of the virus has been associated with more severe acute COVID-19,[34] so raised EBV-associated-risks may be also be present among those without an IM diagnosis. We speculate that the raised risk of hospital-diagnosed EBV-associated IM following SARS-CoV-2 infection may indicate a 'double-hit' phenomenon, potentially further raising risk of future MS among those exposed to both viral infections in a relatively short space of time.

While this study is both large and comprehensive, and identified all known diagnoses of COVID-19 in Sweden during the follow-up period, there are also some potential limitations. An important limitation is that the only measure of greater COVID-19 severity that we could use was whether the infection resulted in hospital admission. This does not identify specific symptoms of potential importance or other factors than may influence the decision to admit someone with the infection, so there is likely to be some heterogeneity in this putative marker of severity. The number of individuals with the outcome diagnoses of non-MS demyelinating diseases is small, and more than half of them have a non-specific diagnosis, which may question diagnostic accuracy so the results should be interpreted with some caution. This lack of a specific diagnosis, at least in part, may be because of insufficient follow-up time to identify a more specific diagnosis. However, in the context of all demyelinating diagnoses including MS, the non-specific diagnoses are a minority. As diagnostic precision remains an issue and disease

For Review Only

progression is incompletely described, further research should examine evidence of progression of demyelination with information on diagnostic methods, including use of repeated MRI examinations. The results are consistent across the outcomes and signal the plausibility that other members of the population may have as-yet undiagnosed demyelinating disease. Many further individuals may have been infected but were not tested by PCR (however, testing during the major period of the study period was extensive, although there was limited testing at the beginning of the pandemic). Some demyelinating diseases, particularly MS, are insidious in onset, with development over a long period and so will mostly not have been detected during the limited follow-up time of this study. The SARS-CoV-2 viral variant responsible for the infection could not be identified in our data so calendar period was used as the underlying timescale in the analysis to take aspects of the changing characteristics of the pandemic into account. We also stratified by period of exposure: up to the end of 2020 and then subsequently. Shared susceptibility to SARS-CoV-2 infection and the outcomes could explain some of the associations. To address this, anyone with a demyelinating diagnosis prior to 2020 was excluded and we adjusted for and stratified by Charlson comorbidity index score to control for underlying health problems: as expected, a higher Charlson comorbidity index score was generally positively associated with the outcomes, but the demyelinating outcomes associated with COVID-19 were found most notably among those without comorbidity. Some results may have been influenced by surveillance/referral bias due to concern about symptoms of infection among those with a hospital admission for COVID-19.

We found an increased risk of demyelinating diseases of the CNS among people treated in hospital for COVID-19, as well as a raised risk of EBV-related infectious mononucleosis, an established risk factor for MS. At least a proportion of these associations with COVID-19 may potentially be due to shared susceptibility or surveillance bias. The occurrence of delayed-onset

For Review Only

demyelinating diseases such as MS should continue to be assessed among those who experienced COVID-19, as MS can have long asymptomatic and prodromal phases.

Data availability

The data used in this study are deidentified individual level data from Swedish healthcare registers and can be obtained from the respective Swedish public data holders with ethical approval for the research in question, subject to relevant legislation, processes, and data protection.

Funding

This study was funded by grants from *Nyckelfonden*. The SCIFI-PEARL project has basic funding based on grants from the Swedish state under the agreement between the Swedish government and the county councils, the ALF-agreement (*Avtal om Läkarutbildning och Forskning*/Medical Training and Research Agreement) grants ALFGBG-938453, ALFGBG-971130, ALFGBG-978954, and previously from a joint grant from FORTE (*Forskningsrådet för hälsa, arbetsliv och* välfärd/Research Council for Health, Working Life, and Welfare) and FORMAS (*Forskningsrådet för miljö, areella näringar och samhällsbyggande*/Research Council for Environment, Agricultural Sciences and Spatial Planning), grant 2020-02828.

Competing interests

SM has received MS research grants and/or honoraria for advisory boards/lectures from Roche, Novartis, AstraZeneca, Merck, Teva and IQVIA. FN owns AstraZeneca shares. The other authors have no disclosures.

For Review Only

For Review Only

References

1. Lanz TV, Brewer RC, Ho PP, et al. Clonally expanded B cells in multiple sclerosis bind EBV EBNA1 and GlialCAM. *Nature* 2022 doi: 10.1038/s41586-022-04432-7 [published Online First: 2022/01/25]
2. Bjornevik K, Cortese M, Healy BC, et al. Longitudinal analysis reveals high prevalence of Epstein-Barr virus associated with multiple sclerosis. *Science* 2022;375(6578):296-301. doi: 10.1126/science.abj8222 [published Online First: 2022/01/14]
3. Xu Y, Hiyoshi A, Smith KA, et al. Association of Infectious Mononucleosis in Childhood and Adolescence With Risk for a Subsequent Multiple Sclerosis Diagnosis Among Siblings. *JAMA Netw Open* 2021;4(10):e2124932. doi: 10.1001/jamanetworkopen.2021.24932 [published Online First: 2021/10/12]
4. Arino H, Heartshorne R, Michael BD, et al. Neuroimmune disorders in COVID-19. *J Neurol* 2022;269(6):2827-39. doi: 10.1007/s00415-022-11050-w [published Online First: 2022/03/31]
5. Giovannoni G. How long is the presymptomatic phase of multiple sclerosis? *Mult Scler Relat Disord* 2016;7:12-3. doi: 10.1016/j.msard.2016.02.010 [published Online First: 2016/05/31]
6. Xu Y, Smith KA, Hiyoshi A, et al. Hospital-diagnosed infections before age 20 and risk of a subsequent multiple sclerosis diagnosis. *Brain* 2021;144(8):2390-400. doi: 10.1093/brain/awab100 [published Online First: 2021/03/12]
7. Optic Neuritis Study G. Multiple sclerosis risk after optic neuritis: final optic neuritis treatment trial follow-up. *Arch Neurol* 2008;65(6):727-32. doi: 10.1001/archneur.65.6.727 [published Online First: 2008/06/11]
8. Ismail, II, Salama S. Association of CNS demyelination and COVID-19 infection: an updated systematic review. *J Neurol* 2022;269(2):541-76. doi: 10.1007/s00415-021-10752-x [published Online First: 2021/08/14]
9. Correale J, Fiol M, Gilmore W. The risk of relapses in multiple sclerosis during systemic infections. *Neurology* 2006;67(4):652-9. doi: 10.1212/01.wnl.0000233834.09743.3b [published Online First: 2006/07/28]
10. Ryan FJ, Hope CM, Masavuli MG, et al. Long-term perturbation of the peripheral immune system months after SARS-CoV-2 infection. *BMC Med* 2022;20(1):26. doi: 10.1186/s12916-021-02228-6 [published Online First: 2022/01/15]
11. Nouri MN YE. Neuroinflammatory and Demyelinating Disorders of Childhood. *Clinical Child Neurology* 2020;9:651-77.
12. Nyberg F, Franzen S, Lindh M, et al. Swedish COVID-19 Investigation for Future Insights - A Population Epidemiology Approach Using Register Linkage (SCIFI-PEARL). *Clin Epidemiol* 2021;13:649-59. doi: 10.2147/CLEP.S312742 [published Online First: 2021/08/07]
13. Charlson ME, Pompei P, Ales KL, MacKenzie CR. A new method of classifying prognostic comorbidity in longitudinal studies: development and validation. *J Chronic Dis* 1987;40(5):373-83. doi: 10.1016/0021-9681(87)90171-8 [published Online First: 1987/01/01]
14. Quan H, Li B, Couris CM, et al. Updating and validating the Charlson comorbidity index and score for risk adjustment in hospital discharge abstracts using data from 6 countries. *Am J Epidemiol* 2011;173(6):676-82. doi: 10.1093/aje/kwq433 [published Online First: 2011/02/19]

For Review Only

15. Quan H, Sundararajan V, Halfon P, et al. Coding algorithms for defining comorbidities in ICD-9-CM and ICD-10 administrative data. *Med Care* 2005;43(11):1130-9. doi: 10.1097/01.mlr.0000182534.19832.83 [published Online First: 2005/10/15]
16. Moore L, Ghannam M, Manousakis G. A first presentation of multiple sclerosis with concurrent COVID-19 infection. *eNeurologicalSci* 2021;22:100299. doi: 10.1016/j.ensci.2020.100299 [published Online First: 2020/12/15]
17. Yavari F, Raji S, Moradi F, Saeidi M. Demyelinating Changes Alike to Multiple Sclerosis: A Case Report of Rare Manifestations of COVID-19. *Case Rep Neurol Med* 2020;2020:6682251. doi: 10.1155/2020/6682251 [published Online First: 2021/01/12]
18. Smith KA, Hiyoshi A, Burkill S, et al. Hospital diagnosed pneumonia before age 20 years and multiple sclerosis risk. *BMJ Neurol Open* 2020;2(1):e000044. doi: 10.1136/bmjno-2020-000044 [published Online First: 2021/03/09]
19. Loebermann M, Winkelmann A, Hartung HP, et al. Vaccination against infection in patients with multiple sclerosis. *Nat Rev Neurol* 2012;8(3):143-51. doi: 10.1038/nrneurol.2012.8 [published Online First: 2012/01/25]
20. Liang Y, Hou L, Hou G, et al. The influences of the COVID-19 pandemic on Epstein-Barr virus infection in children, Henan, China. *J Infect* 2023;86(5):525-28. doi: 10.1016/j.jinf.2023.01.021 [published Online First: 2023/01/24]
21. Kurra N, Woodard PI, Gandrakota N, et al. Opportunistic Infections in COVID-19: A Systematic Review and Meta-Analysis. *Cureus* 2022;14(3):e23687. doi: 10.7759/cureus.23687 [published Online First: 2022/05/05]
22. Montgomery S, Hiyoshi A, Burkill S, et al. Concussion in adolescence and risk of multiple sclerosis. *Ann Neurol* 2017;82(4):554-61. doi: 10.1002/ana.25036 [published Online First: 2017/09/05]
23. Montgomery S, Hiyoshi A, Burkill S, et al. Reply to "concussion may not cause multiple sclerosis". *Ann Neurol* 2017;82(4):652-53. doi: 10.1002/ana.25062 [published Online First: 2017/10/05]
24. Rogers JP, Watson CJ, Badenoch J, et al. Neurology and neuropsychiatry of COVID-19: a systematic review and meta-analysis of the early literature reveals frequent CNS manifestations and key emerging narratives. *J Neurol Neurosurg Psychiatry* 2021;92(9):932-41. doi: 10.1136/jnnp-2021-326405 [published Online First: 2021/06/05]
25. Murray RS, Cai GY, Hoel K, et al. Coronavirus infects and causes demyelination in primate central nervous system. *Virology* 1992;188(1):274-84. doi: 10.1016/0042-6822(92)90757-g [published Online First: 1992/05/01]
26. Desforges M, Le Coupanec A, Dubeau P, et al. Human Coronaviruses and Other Respiratory Viruses: Underestimated Opportunistic Pathogens of the Central Nervous System? *Viruses* 2019;12(1) doi: 10.3390/v12010014 [published Online First: 2019/12/22]
27. Cao X. COVID-19: immunopathology and its implications for therapy. *Nat Rev Immunol* 2020;20(5):269-70. doi: 10.1038/s41577-020-0308-3 [published Online First: 2020/04/11]
28. Kostic M, Dzopalic T, Zivanovic S, et al. IL-17 and glutamate excitotoxicity in the pathogenesis of multiple sclerosis. *Scand J Immunol* 2014;79(3):181-6. doi: 10.1111/sji.12147 [published Online First: 2014/01/05]

For Review Only

29. Odoardi F, Sie C, Streyl K, et al. T cells become licensed in the lung to enter the central nervous system. *Nature* 2012;488(7413):675-9. doi: 10.1038/nature11337 [published Online First: 2012/08/24]
30. Tzartos JS, Friese MA, Craner MJ, et al. Interleukin-17 production in central nervous system-infiltrating T cells and glial cells is associated with active disease in multiple sclerosis. *Am J Pathol* 2008;172(1):146-55. doi: 10.2353/ajpath.2008.070690 [published Online First: 2007/12/25]
31. Cepok S, Zhou D, Srivastava R, et al. Identification of Epstein-Barr virus proteins as putative targets of the immune response in multiple sclerosis. *J Clin Invest* 2005;115(5):1352-60. doi: 10.1172/JCI23661 [published Online First: 2005/04/21]
32. Lindsey JW. Antibodies to the Epstein-Barr virus proteins BFRF3 and BRRF2 cross-react with human proteins. *J Neuroimmunol* 2017;310:131-34. doi: 10.1016/j.jneuroim.2017.07.013 [published Online First: 2017/08/06]
33. Baker D, Pryce G, Amor S, et al. Learning from other autoimmunities to understand targeting of B cells to control multiple sclerosis. *Brain* 2018;141(10):2834-47. doi: 10.1093/brain/awy239 [published Online First: 2018/09/14]
34. Im JH, Nahm CH, Je YS, et al. The effect of Epstein-Barr virus viremia on the progression to severe COVID-19. *Medicine (Baltimore)* 2022;101(18):e29027. doi: 10.1097/MD.0000000000029027 [published Online First: 2022/05/14]

For Review Only

Table 1. Baseline characteristics of the study population (N=9 981 915) aged between 3 and 100 years in January 2020, by subsequent SARS-CoV-2 infection status by 30th November 2022

	No diagnosed infection<br>Total n=7,498,492<br>N (%)	Positive SARS-CoV-2 test only<br>Total n=2,371,402<br>N (%)	Hospital admission due to SARS-CoV-2<br>Total n=112,021)<br>N (%)
**Age** (years)			
3-10	716 034 (9.6)	205 879 (8.7)	794 (0.7)
11-20	851 939 (11.4)	323 550 (13.6)	1718 (1.5)
21-30	926 137 (12.4)	410 063 (17.3)	5174 (4.6)
31-40	908 194 (12.1)	444 038 (18.7)	7343 (6.6)
41-50	868 125 (11.58)	421 281 (17.8)	10 194 (9.1)
51-60	960 263 (12.8)	317 894 (13.4)	15 874 (14.2)
61-70	958 268 (12.8)	130 613 (5.5)	18 617 (16.6)
71-80	882 771 (11.8)	63 011 (2.7)	26 547 (23.7)
81-90	357 146 (4.8)	41 226 (1.7)	20 809 (18.6)
91-100	69 615 (0.9)	13 847 (0.6)	4951 (4.4)
**Sex**			
Male	3 856 628 (51.4)	1 100 319 (46.4)	62 231 (55.6)
Female	3 641 864 (48.6)	1 271 083 (53.6)	49 790 (44.5)
**Charlson Comorbidity Index**			
0	6 561 231 (87.5)	2 166 234 (91.4)	62 858 (56.1)
1	358 769 (4.8)	101 845 (4.3)	12 966 (11.6)
2	372 423 (5.0)	71 989 (3.0)	16 980 (15.2)
3 or more	206 069 (2.8)	31 334 (1.3)	19 217 (17.2)
**Region of Sweden**			
North	668 495 (8.9)	180 217 (7.6)	8151 (7.3)
South	1 335 440 (17.8)	436 487 (18.4)	16 198 (14.5)
Stockholm	1 723 729 (23.0)	554 305 (23.4)	31 734 (28.3)
South East	783 644 (10.5)	230 028 (9.7)	11 224 (10.0)
Uppsala-Örebro	1 500 674 (20.0)	502 513 (21.2)	20 488 (18.3)
West	1 350 693 (18.0)	461 271 (19.5)	17 054 (15.2)
Other	135 817 (1.8)	6 581 (0.3)	7 172 (6.4)
**Country of origin**			
Africa	180 238 (2.4)	48 191 (2.0)	3 044 (2.7)
Asia	569 864 (7.6)	200 446 (8.5)	12 766 (11.4)
European Union excluding Nordic countries	297 723 (4.0)	77 320 (3.3)	4 408 (3.9)
Europe excluding European Union and Nordic countries	185 408 (2.5)	76 236 (3.2)	5 776 (5.2)
North America	31 692 (0.4)	9 259 (0.4)	393 (0.4)
Nordic countries excluding Sweden	189 343 (2.5)	36 599 (1.5)	4980 (4.5)
Oceania	4 998 (0.1)	1353 (0.1)	19 (0.0)
Former Soviet Union	4 199 (0.1)	1066 (0.0)	121 (0.1)
Sweden	5 981 516 (79.8)	1 899 892 (80.1)	79 093 (70.6)
South America	52 119 (0.7)	20 763 (0.9)	1408 (1.3)
Other	1 392 (0.0)	277 (0.0)	13 (0.0)
**Demyelinating disease diagnoses prior to 2020**			
No	7 483 154 (99.8)	2 366 117 (99.8)	111 106 (99.2)
Yes	15 338 (0.2)	5285 (0.2)	915 (0.8)

For Review Only

Table 2. Hazard ratios (HR) with 95% confidence intervals (CI) for non-MS demyelinating diseases associated with SARS-CoV-2 status

	N non-MS demyelinating diseases/N total	Rate† (95% CI)	Unadjusted HR (95% CI)	P	Adjusted** HR (95% CI)	P
**Total**	1139/9 959 818	4.0 (3.8 to 4.2)				
**SARS-CoV-2***						
Not diagnosed	969/9 959 776	3.8 (3.6 to 4.1)	Reference		Reference	
Positive test only	158/2 386 881	5.3 (4.5 to6.2)	1.32 (1.11 to1.58)	0.002	1.07 (0.90 to1.29)	0.434
Hospital admission	12/113 574	9.0 (5.1 to15.9)	2.28 (1.29 to 4.04)	0.005	2.31 (1.30 to 4.10)	0.004
**Sex**						
Male	388/5 012 336	2.7 (2.5 to3.0)	Reference		Reference	
Female	751/4 947 482	5.3 (4.9to5.7)	1.96 (1.73 to 2.21)	<0.001	2.02 (1.78 to 2.28)	<0.001
**Charlson Comorbidity Index**						
0	981/8 772 348	3.9 (3.6 to 4.1)	Reference		Reference	
1	60/472 485	4.5 (3.5 to5.7)	1.15 (0.88 to 1.49)	0.298	1.51 (1.16 to 1.96)	0.002
2	73/459 256	5.9 (4.7 to7.4)	1.52 (1.20 to 1.93)	0.001	2.29 (1.79 to 2.93)	<0.001
3 or more	25/255 729	4.3 (2.9 to6.3)	1.11 (0.75 to 1.65)	0.604	1.86 (1.24 to 2.80)	0.003

*SARS-CoV-2 was modelled as a time-varying exposure, hence the total sum of number of observations (individuals) contributing time at risk in each category of SARS-CoV-2 exposure is greater than the total number of individuals.

** Adjusted for birth year (1920-1940, 1941-1960, 1961-1980, 1981-2000, 2001-2016), sex (male, female), regions of Sweden (North, South, Stockholm, South East, Uppsala-Örebro, West, other), country of origin (Africa, Asia, European Union excluding Nordic countries, Europe excluding European Union and Nordic countries, North America, Nordic countries excluding Sweden, Oceania, former Soviet Union, Sweden, South America, other), and Charlson comorbidity index.

†Per 100,000 person-years.

For Review Only

Table 3. Demyelinating diseases (excluding MS), by SARS-CoV-2 infection status by November 2022

**ICD-10**		**Not diagnosed, n=969**	**Positive SARS-CoV-2 test, only n=158**	**Hospital admission, n=12**
**G36**	**Other acute disseminated demyelination**			
G36.0	Neuromyelitis optica [Devic]	89 (8.8%)	11 (6.7%)	2 (16.7%)
G36.1	Acute and subacute haemorrhagic leukoencephalitis [Hurst]	-	1 (0.6%)	-
G36.8	Other specified acute disseminated demyelination	9 (0.9%)	4 (2.4%)	1 (8.3%)
G36.9	Acute disseminated demyelination, unspecified	23 (2.3%)	3 (1.8%)	-
**G37**	**Other demyelinating diseases of central nervous system**			
G37.0	Diffuse sclerosis	4 (0.4%)	1 (0.6%)	-
G37.1	Central demyelination of corpus callosum	4 (0.4%)	-	1 (8.3%)
G37.2	Central pontine myelinolysis	38 (3.8%)	1 (0.6%)	-
G37.3	Acute transverse myelitis in demyelinating disease of central nervous system	32 (3.2%)	2 (1.2%)	-
G37.4	Subacute necrotizing myelitis	2 (0.2%)	1 (0.6%)	-
G37.5	Concentric sclerosis [Baló]	1 (0.1%)	-	-
G37.8	Other specified demyelinating diseases of central nervous system	68 (6.7%)	12 (7.3%)	1 (8.3%)
G37.9	Demyelinating disease of central nervous system, unspecified	740 (73.3%)	129 (78.2%)	7 (58.3%)

Some individuals had more than one demyelinating disease diagnosis.
The percentages indicate proportions only among those with a demyelinating disease and cannot be used to estimate relative risk from this table alone.

For Review Only

Table 4. Hazard ratios (HR) with 95% confidence intervals (CI) for multiple sclerosis associated with SARS-CoV-2 status

	N multiple sclerosis/ N total	Rate† (95% CI)	Unadjusted HR (95% CI)	P	Adjusted** HR (95% CI)	P
**Total**	2787/9 959 816	9.8 (9.4 to 10.2)				
**SARS-CoV-2***						
Not diagnosed	2403/9 959 774	9.5 (9.1 to 9.9)	Reference		Reference	
Positive test only	356/2 386 557	11.9 (11.7 to 13.2)	1.30 (1.16 to 1.47)	<0.001	1.08 (0.96 to1.22)	0.627
Hospital admission	28/113 507	21.0 (14.5 to 30.5)	2.27 (1.57 to 3.30)	<0.001	2.48 (1.70 to 3.61)	<0.001
**Sex**						
Male	926/5 012 335	6.5 (6.1 to 6.9)	Reference		Reference	
Female	1861/4 947 481	13.2 (12.6 to 13.8)	2.03 (1.88 to2.20)	<0.001	2.08 (1.92 to 2.25)	<0.001
**Charlson Comorbidity Index**						
0	2470/8 772 346	9.8 (9.4 to 10.2)	Reference		Reference	
1	120/472 485	8.9 (7.5 to 10.7)	0.91 (0.76 to 1.10)	0.323	1.14 (0.95 to 1.37)	0.157
2	149/459 256	12.0 (10.2 to 14.1)	1.23 (1.04 to 1.45)	0.015	1.60 (1.35 to 1.90)	<0.001
3 or more	48/255 729	8.2 (6.2 to 10.9)	0.84 (0.63 to 1.12)	0.227	1.15 (0.86 to 1.55)	0.342

*SARS-CoV-2 was modelled as a time-varying exposure, hence the total number of observations in each category of SARS-CoV-2 exposure is greater than the total number of individuals.
** adjusted for birth year (1920-1940, 1941-1960, 1961-1980, 1981-2000, 2001-2016), sex (male, female), regions of Sweden (North, South, Stockholm, South East, Uppsala-Örebro, West, other), country of origin (Africa, Asia, European Union excluding Nordic countries, Europe excluding European Union and Nordic countries, North America, Nordic countries excluding Sweden, Oceania, former Soviet Union, Sweden, South America, other), and Charlson comorbidity index.
†Per 100,000 person-years.

For Review Only

Table 5. Hazard ratios (HR) with 95% confidence intervals (CI) for hospital-diagnosed infectious mononucleosis caused by Epstein-Barr virus associated with SARS-CoV-2 status

	N infectious mononucleosis/ N total	Rate† (95% CI)	Unadjusted HR (95% CI)	P	Adjusted** HR (95% CI)	P
**Total**	1439/9 956 763	5.1 (4.8 to 5.3)				
**SARS-CoV-2***						
Not diagnosed	1185/9 956 721	4.7 (4.4 to5.0)	Reference		Reference	
Positive test only	240/2 385 784	8.0 (7.1 to 9.1)	1.70 (1.47 to 1.98)	<0.001	1.61 (1.38 to 1.87)	<0.001
Hospital admission	14/113 521	10.5 (6.2 to 17.8)	2.23 (1.31 to 3.78)	0.003	5.63 (3.29 to 9.66)	<0.001
**Sex**						
Male	653/5 010 947	4.6 (4.2 to 4.9)	Reference		Reference	
Female	786/4 945 816	5.6 (5.2 to 6.0)	1.22 (1.10 to 1.35)	<0.001	1.28 (1.16 to1.42)	<0.001
**Charlson Comorbidity Index**						
0	1223/8 769 678	4.8 (4.6 to 5.1)	Reference		Reference	
1	127/472 243	9.4 (7.9 to 11.2)	1.95 (1.62 to 2.34)	<0.001	1.55 (1.29 to 1.86)	<0.001
2	48/459 172	3.9 (2.9 to 5.1)	0.80 (0.60 to 1.07)	0.132	3.57 (2.64 to 4.84)	<0.001
3 or more	41/255 670	7.0 (5.2 to 9.5)	1.46 (1.07 to 1.99)	0.017	7.89 (5.58 to 11.16)	<0.001

*SARS-CoV-2 was modelled as a time-varying exposure, hence the total number of observations in each category of SARS-CoV-2 exposure is greater than the total number of individuals.

** Adjusted for birth year (1920-1940, 1941-1960, 1961-1980, 1981-2000, 2001-2016), sex (male, female), regions of Sweden (North, South, Stockholm, South East, Uppsala-Örebro, West, other), country of origin (Africa, Asia, European Union excluding Nordic countries, Europe excluding European Union and Nordic countries, North America, Nordic countries excluding Sweden, Oceania, former Soviet Union, Sweden, South America, other), and Charlson comorbidity index.

†Per 100,000 person-years.

For Review Only

Table 6. Hazard ratios (HR) with 95% confidence intervals (CI) for each outcome associated with SARS-CoV-2 status, stratified by sex, Charlson comorbidity index, age category, and country of origin.

SARS-CoV-2*	N non-MS demyelinating diseases/N total	Adjusted** HR (95% CI)	P	N multiple sclerosis/ N total	Adjusted** HR (95% CI)	P	N infectious mononucleosis/ N total	Adjusted** HR (95% CI)	P
**Total**	1139/9 959 818			2787/9 959 816			1439/9 956 763		
**Sex**									
**Male**									
Not diagnosed	341/5 012 301	Reference		800/5 012 300	Reference		557/5 010 912	Reference	
Positive test only	42/1 115 033	0.99 (0.71 to 1.39)	0.973	115/1 114 964	1.20 (0.98 to 1.48)	0.079	86/1 114 561	1.29 (1.01 to 1.64)	0.042
Hospital admission	5/64 072	2.33 (0.95 to 5.69)	0.064	11/64 055	2.65 (1.45 to 4.83)	0.002	10/64 065	6.47 (3.39 to 12.36)	<0.001
**Female**									
Not diagnosed	628/4 947 475	Reference		1603/4 947 474	Reference		628/4 945 809	Reference	
Positive test only	116/1 271 848	1.10 (0.89 to1.36)	0.395	241/1 271 593	1.03 (0.89 to 1.19)	0.705	154/1 271 223	1.90 (1.57 to 2.31)	<0.001
Hospital admission	7/49 475	2.23 (1.05 to 4.72)	0.037	17/49 452	2.38 (1.47 to 3.85)	<0.001	4/49 456	4.02 (1.49 to 10.89)	0.006
**Charlson Comorbidity Index**									
**0**									
Not diagnosed	824/8 772 317	Reference		2112/8 772 315	Reference		998/8 769 647	Reference	
Positive test only	149/ 2 171 596	1.11 (0.92 to 1.33)	0.287	337/2 171 303	1.09 (0.97 to 1.24)	0.158	217/2 170 638	1.65 (1.41 to 1.94)	<0.001
Hospital admission	8/63 963	2.65 (1.32 to 5.33)	0.006	21/63 936	2.89 (1.88 to 4.45)	<0.001	8/63 959	7.75 (3.85 to 15.62)	<0.001
**1 or more**									
Not diagnosed	145/1 187 459	Reference		291/1 187 459	Reference		187/1 187 074	Reference	
Positive test only	9/ 215 286	0.63 (0.31 to 1.27)	0.199	19/215 254	0.81 (0.50 to 1.32)	0.405	23/215 146	1.24 (0.78 to 1.98)	0.360
Hospital admission	4/ 49 584	1.77 (0.65 to 4.82)	0.267	7/49 571	1.84 (0.86 to 3.93)	0.114	6/49 562	4.38 (1.91 to 10.06)	<0.001
**Age categories**									
**3-10 years**									
Not diagnosed	7/922 613	Reference		0/922 613	Reference		151/922 220	Reference	
Positive test only	2/205 903	1.49 (0.29 to 7.67)	0.633	0/205 903	-		17/205 757	1.30 (0.75 to 2.24)	0.345
Hospital admission	0/788	-	-	0/788	-		1/785	8.17 (1.08 to 61.55)	0.042
**11-30 years**									
Not diagnosed	256/2 516 907	Reference		655/2 516 907	Reference		858/2 514 623	Reference	
Positive test only	58/732 313	1.21 (0.89 to 1.64)	0.216	122/732 207	1.12 (0.91 to 1.37)	0.294	196/731 362	1.44 (1.21 to 1.70)	<0.001

For Review Only

Hospital admission	3/6 911	4.41 (1.40 to 13.9)	0.011	6/6 906	4.28 (1.91 to 9.60)	<0.001	4/6895	4.42 (1.65 to 11.87)	0.003
**31-50 years**									
Not diagnosed	397/2 651 144	Reference		976/2 651 143	Reference		80/2 650 877	Reference	
Positive test only	81/866 567	1.11 (0.85 to 1.44)	0.442	178/866 402	1.03 (0.87 to 1.23)	0.727	18/866 570	1.39 (0.80 to 2.43)	0.243
Hospital admission	4/18 030	2.43 (0.90 to 6.54)	0.079	6/18 019	1.55 (0.69 to 3.47)	0.285	2/18 026	4.41 (1.06 to 18.34)	0.042
**51-70 years**									
Not diagnosed	263/2 392 483	Reference		559/2 392 483	Reference		58/2 392 402	Reference	
Positive test only	15/456 787	0.57 (0.33 to 0.97)	0.037	43/456 746	0.79 (0.58 to 1.09)	0.158	7/456 787	1.45 (0.64 to 3.29)	0.376
Hospital admission	4/36 369	1.72 (0.64 to 4.67)	0.285	8/36 359	2.10 (1.04 to 4.25)	0.039	3/36 366	4.12 (1.25 to 13.56)	0.02
**71-100 years**									
Not diagnosed	46/1 476 629	Reference		213/1 476 628	Reference		38/1 476 599	Reference	
Positive test only	2/125 311	1.21 (0.29 to 5.06)	0.796	13/125 299	2.54 (1.43 to 4.52)	0.001	2/125 308	1.66 (0.39 to 7.07)	0.490
Hospital admission	1/51 449	1.37 (0.18 to 10.11)	0.761	8/51 435	4.35 (2.12 to 8.94)	<0.001	4/51 449	6.70 (2.30 to 19.53)	<0.001
**Country of origin**									
**Sweden**									
Not diagnosed	781/7 941 465	Reference		1984/7 941 463	Reference		1109/7 938 578	Reference	
Positive test only	116/1 905 757	0.97 (0.79 to 1.20)	0.787	275/1 905 478	1.01 (0.88 to 1.15)	0.928	228/1 904 681	1.59 (1.36 to 1.86)	<0.001
Hospital admission	8/79 107	2.36 (1.17 to 4.76)	0.017	22/79 077	2.77 (1.81 to 4.23)	<0.001	13/79 082	6.20 (3.54 to 10.85)	<0.001
**Other European**									
Not diagnosed	102/875 980	Reference		209/875 980	Reference		34/875 915	Reference	
Positive test only	19/193 826	1.20 (0.71 to 2.02)	0.503	35/193 805	1.26 (0.86 to 1.85)	0.238	5/193 811	1.75 (0.63 to 4.82)	0.280
Hospital admission	2/15 592	2.30 (0.56 to 9.50)	0.249	2/15 587	1.44 (0.35 to 5.85)	0.610	0/15 594	-	-
**Africa**									
Not diagnosed	20/231 341	Reference		27/231 341	Reference		7/231 333	Reference	
Positive test only	5/48 895	1.61 (0.58 to 4.45)	0.361	6/48 895	2.04 (0.79 to 5.26)	0.142	1/48 896	1.72 (0.20 to 14.84)	0.624
Hospital admission	1/3254	5.09 (0.66 to 39.06)	0.117	2/3254	10.27 (2.32 to 45.41)	0.002	1/3252	12.80 (1.37 to 119.93)	0.026
**Asia**									
Not diagnosed	58/782 096	Reference		159/782 096	Reference		24/782 015	Reference	
Positive test only	16/205 116	1.90 (1.04 to 3.45)	0.036	36/205 095	1.53 (1.04 to 2.26)	0.031	5/205 112	1.86 (0.66 to 5.21)	0.239
Hospital admission	1/13 533	1.56 (0.21 to 11.48)	0.665	2/13 529	1.39 (0.34 to 5.66)	0.647	0/13 533	-	-
**Other*** **									
Not diagnosed	8/128 894	Reference		24/128 894	Reference		11/128 880	Reference	
Positive test only	2/33 287	1.52 (0.27 to 8.56)	0.632	4/33 284	1.10 (0.35 to 3.44)	0.865	1/33 284	0.95 (0.11 to 8.44)	0.963

For Review Only

Hospital admission	0/2061	-	-	0/2060	-	-	0/2060	-	-

*SARS-CoV-2 was modelled as a time-varying exposure, hence the total number of observations in each category of SARS-CoV-2 exposure is greater than the total number of individuals.
** adjusted for birth year (1920-1940, 1941-1960, 1961-1980, 1981-2000, 2001-2016), sex (male, female), regions of Sweden (North, South, Stockholm, South East, Uppsala-Örebro, West, other), country of origin (Africa, Asia, European Union excluding Nordic countries, Europe excluding European Union and Nordic countries, North America, Nordic countries excluding Sweden, Oceania, former Soviet Union, Sweden, South America, other), and Charlson comorbidity index. Each analysis excludes adjustment variable directly corresponding to stratification variable.
***Other countries include North America, Oceania, former Soviet Union, and South America due to low number of outcomes.

For Review Only

Table 7. Hazard ratios (HR) with 95% confidence intervals (CI) for non-MS demyelinating disease, multiple sclerosis, and infectious mononucleosis outcomes associated with SARS-CoV-2 status before and from 1st January, 2021.

SARS-CoV-2*	N non-MS demyelinating diseases/N total	Adjusted** HR (95% CI)	P	N multiple sclerosis/ N total	Adjusted** HR (95% CI)	P	N infectious mononucleosis/ N total	Adjusted** HR (95% CI)	P
**Total**	1139/9 959 818			2787/9 959 816			1439/9 956 763		
Not diagnosed	969/9 959 892	Reference		2403/9 959 890	Reference		1185/9 956 837	Reference	
Positive test only before 01/01/2021	43/438 385	0.97 (0.71 to 1.33)	0.862	112/438 355	1.10 (0.91 to 1.34)	0.321	46/438 202	1.40 (1.04 to 1.89)	0.027
Positive test only after 01/01/2021	115/1 949 075	1.12 (0.91 to 1.38)	0.280	244/ 1 948 781	1.07 (0.93 to 1.23)	0.332	194/1 948 161	1.68 (1.42 to 1.99)	<0.001
Hospital admission before 01/01/2021	7/42 042	2.57 (1.22 to 5.44)	0.013	11/42 029	1.86 (1.03 to 3.38)	0.040	3/42 024	2.31 (0.74 to 7.23)	0.150
Hospital admission after 01/01/2021	5/70 810	2.04 (0.84 to 4.92)	0.114	17/70 783	3.18 (1.97 to 5.13)	<0.001	11/70 802	9.35 (5.11 to 17.10)	<0.001

*SARS-CoV-2 was modelled as a time-varying exposure, hence the total number of observations in each category of SARS-CoV-2 exposure is greater than the total number of individuals.
** Adjusted for birth year (1920-1940, 1941-1960, 1961-1980, 1981-2000, 2001-2016), sex (male, female), regions of Sweden (North, South, Stockholm, South East, Uppsala-Örebro, West, other), country of origin (Africa, Asia, European Union excluding Nordic countries, Europe excluding European Union and Nordic countries, North America, Nordic countries excluding Sweden, Oceania, former Soviet Union, Sweden, South America, other), and Charlson comorbidity index.

For Review Only
