# Supplementary material for: SARS-CoV-2 infection and risk of subsequent demyelinating diseases: national register–based cohort study
Source: Brain Commun. 2024 Nov 29;6(6):fcae406. doi: 10.1093/braincomms/fcae406 (PMC11629974; doi:10.1093/braincomms/fcae406)
Supplement: fcae406_Supplementary_Data [file fcae406_supplementary_data.zip › Revision_1_manuscript.pdf]

OXFORD UNIVERSITY PRESS | Brain Communications

SARS-CoV-2 infection and risk of subsequent demyelinating diseases: national register-based cohort study

Journal:	*Brain Communications*
Manuscript ID	BRAINCOM-2024-038.R1
Manuscript Type:	Original Article
Date Submitted by the Author:	09-Jun-2024
Complete List of Authors:	Montgomery, Scott; Örebro University; Karolinska Institutet, Department of Medicine; University College London, Department of Epidemiology and Public Health<br>Vingeliene, Snieguole; Örebro University<br>Li, Huiqi; University of Gothenburg<br>Backman, Helena; Örebro University, Department of Obstetrics and Gynaecology<br>Udumyan, Ruzan; Örebro University<br>Jendeberg, Johan; Örebro University, Department of Radiology<br>Rasmussen, Gunlög; Örebro University, Department of Infectious Diseases<br>Sundqvist, Martin; Örebro University, Department of Laboratory Medicine Clinical Microbiology<br>Fall, Katja; Örebro University; Karolinska Institutet<br>Hiyoshi, Ayako; Örebro University<br>Nyberg, Fredrik; University of Gothenburg, School of Public Health and Community Medicine, Institute of Medicine, Sahlgrenska Academy
Keywords:	SARS-CoV-2, multiple sclerosis, demyelinating disease

SCHOLARONE™
Manuscripts

SARS-CoV-2 infection and risk of subsequent demyelinating diseases: national register-based cohort study

Short title: COVID-19 and demyelinating disease

Scott Montgomery, professor, scott.montgomery@oru.se (0000-0001-6328-5494),[1 2 3]
Snieguole Vingeliene, epidemiologist, snieguole.vingeliene@oru.se,[1]
Huiqi Li, associate professor, huiqi.li@gu.se,[4]
Helena Backman, associate professor, Helena.Backman@oru.se (0000-0002-2691-7525)[5]
Ruzan Udumyan, epidemiologist, ruzan.udumyan@regionorebrolan.se,[1]
Johan Jendeberg, radiologist, johan.jendeberg@regionorebrolan.se (0000-0001-8949-119X)[6]
Gunlög Rasmussen, senior consultant, gunlog.rasmussen@regionorebrolan.se,[7]
Martin Sundqvist, senior consultant, Martin.Sundqvist@regionorebrolan.se,[8]
Katja Fall, professor, Katja.Fall@oru.se,[1 9]
Ayako Hiyoshi, associate professor, ayako.hiyoshi@oru.se (0000-0002-2088-0530),[1]
Fredrik Nyberg, professor, fredrik.nyberg.2@gu.se (0000-0003-0892-5668)[4]

1. Clinical Epidemiology and Biostatistics, School of Medical Sciences, Faculty of Medicine and Health, Örebro University, Örebro, Sweden.
2. Clinical Epidemiology Division, Department of Medicine, Solna, Karolinska Institutet, Stockholm, Sweden.
3. Department of Epidemiology and Public Health, University College London, London, UK.
4. School of Public Health and Community Medicine, Institute of Medicine, Sahlgrenska Academy, University of Gothenburg, Gothenburg, Sweden.
5. Department of Obstetrics and Gynaecology, Faculty of Medicine and Health, Örebro University, Örebro, Sweden.
6. Department of Radiology, Faculty of Medicine and Health, Örebro University, Örebro, Sweden.
7. Department of Infectious Diseases, School of Medical Sciences, Faculty of Medicine and Health, Örebro University, Örebro, Sweden.
8. Department of Laboratory Medicine, Clinical Microbiology, Faculty of Medicine and Health, Örebro University, Örebro, Sweden.
9. The Institute of Environmental Medicine, Karolinska Institutet, Stockholm, Sweden

Correspondence to
Prof. Scott Montgomery
Clinical Epidemiology and Biostatistics
School of Medical Sciences
Faculty of Medicine and Health
Örebro University
Campus USÖ
Södra Grev Rosengatan 30
703 62 Örebro
Sweden

Telephone +46 727236210

For Review Only

Abstract

Demyelinating diseases, including multiple sclerosis, are associated with prior infectious exposures, so we assessed whether SARS-CoV-2 infection is associated with subsequent diagnoses of non-multiple sclerosis demyelinating diseases and multiple sclerosis. All residents of Sweden aged 3 to 100 years were followed between 1st January 2020 and 30th November 2022, excluding those with demyelinating disease prior to 2020, comprising 9,981,915 individuals divided into uninfected, and those who were infected were categorised into those with and without hospital admission for the infection as a marker of infection severity. Cox regression assessed the risk of two separate outcomes: hospital diagnosed non-multiple sclerosis demyelinating diseases of the central nervous system and multiple sclerosis. The exposures were modelled as time-varying covariates (uninfected, infection without hospital admission and infected with hospital admission). Hospital admission for COVID-19 was associated with raised risk of subsequent non-multiple sclerosis demyelinating disease, but only 12 individuals had this outcome among the exposed, and of those seven has an unspecified demyelinating disease diagnosis. Rates per 100 000 person years (and 95% confidence intervals) were 3.8 (3.6 to 4.1) among those without a COVID-19 diagnosis and 9.0 (5.1 to 15.9) among those admitted to hospital for COVID-19, with an adjusted hazard ratio and (and 95% confidence interval) of 2.35 (1.32 to 4.18, p=0.004). Equivalent associations with multiple sclerosis (28 individuals had this outcome among the exposed) were rates of 9.5 (9.1 to 9.9) and 21.0 (14.5 to 30.5), and an adjusted hazard ratio of 2.48 (1.70 to 3.61, p<0.001). Hospital admission for COVID-19 was associated with a subsequent raised risk of demyelinating diseases of the central nervous system. Greater duration of follow-up will assist further in differentiating between causal associations and potentially shared susceptibility or surveillance bias for the associations of COVID-19 with

For Review Only

risk of multiple sclerosis and other demyelinating diseases, which can have long asymptomatic and prodromal phases.

Keywords: SARS-CoV-2, multiple sclerosis, demyelinating disease

Abbreviated summary

Montgomery et al. report that hospital admission for COVID-19 was associated with raised risks of subsequent non-multiple sclerosis demyelinating diseases of the CNS and multiple sclerosis. Longer-term follow-up is required to determine if the associations are causal, or have other explanations, such as shared susceptibility or increased contact with healthcare.

For Review Only

Introduction

There is some limited evidence to suggest that SARS-CoV-2 infection may be associated with demyelinating diseases of the central nervous system.[1 2] Given SARS-CoV-2 infection can have consequences for the central nervous system (CNS), including autoimmune disorders,[3] how likely is it that it increases risk of demyelinating diseases of the CNS? This is possibly consistent with the association of some types of viral infections, particularly due to Epstein-Barr virus (EBV), and risk of subsequent multiple sclerosis (MS).[4-6] However, as there can be an extended asymptomatic/prodromal period in MS, and there may even be a 10-to-20-year duration between an acute triggering infection and an MS diagnosis,[6-8] it may be premature to assess fully associations with MS.

Given the relatively short duration since the beginning of the pandemic and the likely extended duration between infection and MS diagnosis,[6-8] associations of infection with a first MS diagnosis may be because the infection represents a precipitating event for an already initiated disease process. While initiation of MS pathogenesis cannot be ruled-out entirely, it may be more likely that a systemic infection causes an exacerbation[9] that leads to an MS diagnosis, so it is useful to consider associations of COVID-19 with MS separately from the other demyelinating diagnoses.

We used healthcare and other register data to study the entire population of Sweden aged three to 100 years from January 2020 to identify associations of COVID-19 with subsequent non-MS demyelinating diseases and MS.

Materials and methods

https://mc.manuscriptcentral.com/braincom

For Review Only

This register-based study included all individuals aged between three and 100 years who were resident in Sweden on 1st of January 2020 (N=9 981 915). This age range was chosen as the more common demyelinating diseases tend to occur after early infancy in children,[10] so children under age three years were excluded as there may be greater diagnostic uncertainty in this group. The data for people over age 100 years are sparse, so the maximum inclusion age was 100 years. The data for the current analysis are part of the SCIFI-PEARL (Swedish COVID-19 Investigation for Future Insights - a Population Epidemiology Approach using Register Linkage) project database, which includes broad sociodemographic and healthcare information on the full general population of Sweden, including all PCR-verified COVID-19 diagnoses.[11] Most data for the study came from national registers and were available from 1st of January 2015 to 30th November 2022 to identify risk factors and temporal trends pre-dating the pandemic. The Total Population Register provided information on dates of birth, death, immigration, and emigration, as well as sex, region of residence and region of birth. SmiNet, the national register of notifiable communicable diseases managed by the Public Health Agency of Sweden, was used to identify all individuals with a positive SARS-CoV-2 PCR test. The Patient Register provided information on hospital diagnoses (particularly at the beginning of the pandemic, there may have been a small number of hospital patients with COVID-19 diagnosis not verified by PCR testing). The Patient Register has existed since 1964 for inpatient care, achieving full national coverage in 1987, with the addition of specialist outpatient diagnoses in 2001. The Swedish Intensive Care Register identifies dates of admission to intensive care (either directly or transferred within the hospital), with data available for this study from 1st January 2020.

Exposures

For Review Only

The marker of more severe SARS-CoV-2 infection was defined as hospital inpatient admission with a diagnosis of COVID-19 (including admission to intensive care, directly or from another inpatient setting) identified using the National Patient Register (NPR) and Intensive Care registers, using the Swedish version of International Classification of Diseases 10 (ICD-10) codes U07.1 or U07.2. Less severe disease was defined as a positive PCR test from SmiNet or an outpatient visit to specialist care with diagnosis codes U07.1 or U07.2 in the NPR, without hospital admission. Where both measures of exposure occurred in the same patient, the marker of more severe disease replaced the less severe in the analysis.

Outcomes

The outcomes studied were hospital outpatient and inpatient diagnoses of non-MS demyelinating disease and MS. The non-MS demyelinating diagnoses were defined using ICD-10 codes G36 and G37 (other acute disseminated demyelination and other demyelinating diseases of central nervous system) from primary or secondary outpatient and inpatient diagnoses recorded in the Patient Register. MS (G35) was examined separately using primary or secondary outpatient and inpatient diagnoses, as the long asymptomatic/prodromal phases seen in MS[6-8] imply that diagnoses shortly after infection may represent an exacerbation of a pre-existing disease processes rather than initiation of pathogenesis.

Covariates

Data from the Patient Register were used to create a Charlson Comorbidity Index[12-14] (categorized as 0, 1, 2, ≥3) comprising myocardial infarction, congestive heart failure, peripheral vascular disease, cerebrovascular disease, chronic pulmonary disease, rheumatic disease, dementia, liver disease, diabetes mellitus, hemiplegia/paraplegia, renal disease, malignancy, metastatic tumours, peptic ulcer disease, and HIV/AIDS. Other variables were

For Review Only

sex; year of birth (1920-1940, 1941-1960, 1961-1980, 1981-2000, 2001-2016); region of birth (Africa, Asia, European Union excluding Nordic countries, Europe excluding European Union and Nordic countries, North America, Nordic countries excluding Sweden, Oceania, former Soviet Union, Sweden, South America, and other); and Swedish healthcare region (North, South, Stockholm, South-East, Uppsala-Örebro, West, and other). Infectious mononucleosis caused by Epstein-Barr virus was considered as a potential mediating factor and was identified as a primary or secondary outpatient or inpatient hospital diagnosis in the Patient Register using ICD-10 code B27.0.

For Review Only

Statistical analysis

The characteristics of the participants were summarized using frequencies and percentages, cross-tabulated by highest severity of COVID-19 infection status as defined above by 30th November 2022. Additional analysis examined outcomes associated with admission to intensive care with COVID-19, compared with the no diagnosis category.

People with a diagnosis of any demyelinating disease, G35, G36, G37 and G040.2 (post-immunization acute disseminated encephalitis, myelitis and encephalomyelitis) prior to 2020 were excluded from the analysis.

The two outcomes were analysed separately: non-MS demyelinating disease and MS. The study population was followed from 1st January 2020 to the first occurrence of the outcome diagnosis, date of emigration, death, or 30th November 2022, whichever occurred first. A time-varying variable changed exposure status in the sequence described in the exposures section, with risk estimated from the first occurrence of the exposure indicator. Calendar time was the underlying time scale as design this will help to address timing of virus waves and implementation of policies designed to limit transmission. Stratification was also performed

by exposure before or from 1st January 2021, as vaccination against SARS-CoV-2 began towards the end of 2020 in Sweden and the Alpha variant was identified as dominant in early 2021, then to be replaced by others. We estimated rates of outcomes of interest per 100 000 person-years with 95% confidence intervals (CI) and used Cox proportional hazards regression to estimate hazard ratios with 95% CI and p-values. Adjustment was for sex, year of birth (age), Charlson Comorbidity Index, healthcare region and region of birth. Kaplan–Meier plots were used to present the duration between hospital admission for COVID-19 and diagnosis of non-MS demyelinating disease and MS.

The Schoenfeld residual test assessed the proportional hazards assumption, which was not violated. Statistical significance was defined as confidence intervals that do not include 1.00 and $p<0.05$.

All analyses were conducted using Stata version 17.0 (StataCorp LLC).

Ethical Approval

Ethical approval for the SCIFI-PEARL project was obtained from the Swedish Ethical Review Authority (2020-01800 with subsequent amendments).

Results

Table 1 shows the characteristics of the Swedish population aged three to 100 years in January 2020. The time-varying SARS-CoV-2 infection status variable is tabulated such that individuals were classified to indicate the most serious infection category by the end of follow-up: someone who tested positive and was later admitted to hospital (with COVID-19, only appears in the hospital admission column, even if they have a subsequent positive PCR test without hospital admission. Those who only tested positive without hospital admission

For Review Only

tended to be younger adults, while those admitted to hospital with COVID-19 were on average older adults (Table 1). Those admitted to hospital were more likely to have pre-existing poorer health, as indicated by the Charlson Comorbidity Index. People with any demyelinating disease before baseline were excluded from all subsequent analysis. During follow-up there were 1139 hospital outpatient or inpatient diagnoses of non-MS demyelinating disease and 2787 of MS.

Non-MS demyelination

Hospital admission for SARS-CoV-2 was associated with a statistically significant raised risk of subsequent non-MS demyelinating disease, both before and after adjustment for potential confounding factors (P=0.004), and adjustment did not attenuate the magnitude of association (table 2). The association with positive test only was attenuated and remained slightly above 1.00 but not statistically significant after adjustment (P=0.541). Females and those with a pre-existing higher Charlson Comorbidity Index score were also more likely to experience demyelinating disease (table 2). The median age at diagnosis of demyelination following hospital admission for COVID-19 was 42 years (range, 25 to 89 years) and median duration between admission for COVID-19 and diagnosis of demyelination was 89 days (range, six to 809 days). Figure 1 (panel A) shows the duration between hospital admission for COVID-19 and non-MS demyelinating disease diagnoses. The majority of these diagnoses were made in the first six months, but one third of them were diagnosed over a year after admission for COVID-19. The specific demyelination diagnoses associated with admission for COVID-19 are shown in Table 3. There was only a small number of individuals with non-MS demyelinating disease outcomes associated with hospital admission for COVID-19 (n=12). The diseases associated with COVID-19 hospital admission were in the following diagnostic categories: neuromyelitis optica; clinically isolated syndrome and other specified acute

For Review Only

disseminated demyelination; central demyelination of the corpus callosum; myelin oligodendrocyte glycoprotein antibody disease and other specified demyelinating diseases of the CNS; and unspecified diseases of the central nervous system.

MS

After adjustment for the potential confounding factors, hospital admission for COVID-19 was associated with raised risk of a first MS diagnosis (P<0.001), but positive test only did not represent a raised risk (table 4). A total of 28 individuals were diagnosed with MS following hospital admission for COVID-19. The association of pre-existing Charlson Comorbidity Index with MS was inconsistent. The median age at MS diagnosis in those admitted to hospital for infection was 56.7 years (range, 15.9 to 89.9 years). In this group, the median duration between admission for COVID-19 and diagnosis of MS was 82 days (range, 5 to 736 days). Figure 1 (panel B) shows the duration between hospital admission for COVID-19 and first MS diagnosis. The majority of these diagnoses were made in the first six months, and only approximately one fifth were diagnosed over a year after admission for COVID-19.

Among people who were admitted to hospital for COVID-19 and who also had a diagnosis of non-MS demyelinating disease or MS, there was no record of hospital-diagnosed infectious mononucleosis between the dates of SARS-CoV-2 infection and diagnosis of demyelinating disease.

Infection before 1st January 2021 and subsequently

When exposure was separated into before and from 1st January 2021, there was no notable difference for non-MS demyelination, but a non-conclusive suggestion of somewhat higher magnitude MS risk associated with hospital admission for COVID-19 after 2020 (Table 5).

For Review Only

Discussion

This national study of SARS-CoV-2 infection found that hospital admission for COVID-19 was associated with an increased risk of non-MS demyelinating diseases, as well as an increased risk of an MS diagnosis.

While this study provides some of the most comprehensive evidence to date that SARS-CoV-2 infection is associated with subsequent demyelinating diseases of the CNS, it should be considered that the association might be explained by shared susceptibility or surveillance bias (medical management of one condition results in diagnosis of another). Earlier research has suggested this association but was based only on small studies with methodological limitations, as reported in a systematic review that summarized evidence of possible associations with encephalomyelitis, brain demyelination, transverse myelitis, neuromyelitis optica and MOG antibody-associated disease.[1] The review further identified a total of three individuals with MS-like demyelination, with symptomatic infection pre-dating onset of neurological symptoms by several weeks in the previous studies.[1 15 16] However, one of these previous studies found the characteristics of the demyelination was not typical for MS, suggesting post-viral demyelination rather than true MS.[1] Another systematic review concluded that CNS demyelinating events associated with SARS-CoV-2 infections occurred at a low rate, consistent with the low rates observed in our study.[2] The current study benefits from follow-up of a national population, with prospectively recorded exposures and greater power to detect rare outcome events.

Our findings are consistent with other research showing that a variety of infections, with likely direct or indirect access to the CNS, are associated with an increased risk of MS.[8 17] The first

https://mc.manuscriptcentral.com/braincom
For Review Only

focus of this study was on demyelination excluding MS, due to the likely long asymptomatic and prodromal periods of MS,[6-8] as there may be insufficient time between infection and frank symptomatic onset of MS in most people who may develop the disease in this population. Despite this, there was a raised risk of new MS diagnoses associated with hospital admission for SARS-CoV-2 infection. The numbers are small, so this may represent a minority of patients with more rapid-onset initiation of MS pathogenesis. An alternative explanation is that the MS disease process had already begun but was undiagnosed in these individuals. This interpretation is consistent with the average age of MS diagnosis that we observed: onset at over 50 years of age suggests that the disease process began some years earlier. The infection may have resulted in an exacerbation and disease progression, such that symptomatic onset led to an MS diagnosis: both bacterial and viral infections are linked with MS exacerbations and disease progression.[18] It is of note that non-MS demyelination diagnoses following hospital admission for COVID-19 tended to occur in earlier adulthood. The majority of diagnoses of both outcomes occurred during the first six months after hospital admission for COVID-19, suggesting precipitation of a pre-existing disease process or diagnoses made due to surveillance resulting from increased contact with healthcare professionals. A minority of the demyelinating disease diagnoses were made over a year after hospital admission for COVID-19, which does not eliminate the possibility of surveillance bias due to continued increased healthcare contact, but also suggests the importance of longer-term follow-up to identify associations with delayed disease onset.

One potential explanation of our findings of raised risks for demyelinating disease is that immune system perturbation is more common after SARS-CoV-2 infection[19] resulting in greater risk of infectious mononucleosis due to Epstein-Barr virus, which is one of the most important environmental risk factors for MS.[4-6] Our results do not support this explanation, as

For Review Only

there were no diagnoses of IM between dates of hospital admission for COVID-19 and diagnosis of non-MS demyelinating disease or MS among those who experienced both exposure and outcomes. However, a potential limitation is that only infectious mononucleosis diagnosed in hospital could be identified.

There is evidence from this study of associations of SARS-CoV-2 infection with CNS demyelination (the extent to which this is a causal association requires more evidence), but there is a question over whether the pandemic will have an influence on future risk beyond what is reported here. There is typically a 10-to-20-year duration between exposure to an environmental risks factor, including acute infections[6-8] and other exposures,[20 21] and an MS diagnosis. It should however be noted that the vast majority of those who go on to develop MS do not have a diagnosed demyelinating event or other evidence of early MS-onset in the years immediately following infections or other environmental exposures linked with MS risk.[6 8 21] Given the possible CNS involvement in SARS-CoV-2 infection,[22] there may be a possibility of future demyelinating disease diagnoses, but perhaps this will be largely among those with more severe COVID-19, as shown previously for other infections with direct or indirect access to the CNS.[6 8 17]

Research using animal models has indicated that corona-virus infections can give rise to immune dysregulation, resulting in MS-like demyelination[23] and are associated with upregulation of a range of proinflammatory cytokines that can cross the blood-brain barrier, creating a highly proinflammatory environment in the CNS.[24] More specifically, increased levels of IL-17 have been observed in coronavirus infected patients[25], which has been implicated in MS aetiology based on animal models.[26] It is also possible that some respiratory infections result in autoreactive T cells in the lungs that enter bronchus-associated lymphoid tissue (BALT) and can then cross the blood-brain barrier, resulting in CNS inflammation.[27]

https://mc.manuscriptcentral.com/braincom
For Review Only

Respiratory infections, likely including COVID-19, can induce a local and systemic T-helper (Th)17 response, including memory Th17 cells, and mechanisms such as molecular mimicry could result in Th17 reactivation and CNS infiltration.[28] Thus, in general lung and BALT have been implicated in pathogenesis of autoimmune neuro-inflammation,[27] and thus indicating the potential plausibility of COVID-19 influencing demyelination risk. The mechanisms linking SARS-CoV-2 with demyelinating disease may share similarities with the mechanisms linking EBV with MS risk. For example, EBV proteins such as BRRF2, EBNA-1, and the small capsid protein, BFRF3, share epitopes with myelin.[29 30] Antibodies to these proteins can cross-react with myelin antigens leading to autoimmune damage to the CNS.[29 30]

While this study is both large and comprehensive, and identified all known diagnoses of COVID-19 in Sweden during the follow-up period, there are also some potential limitations. An important limitation is that the only measure of greater COVID-19 severity that we could use was whether the infection resulted in hospital admission. This does not identify specific symptoms of potential importance or other factors than may influence the decision to admit someone with the infection, so there is likely to be some heterogeneity in this putative marker of severity. The number of individuals with the outcome diagnoses of non-MS demyelinating diseases is small, and more than half of them have a non-specific diagnosis, which may question diagnostic accuracy so the results should be interpreted with caution. The Swedish ICD codes for the outcome diagnoses were limited to one digit after the decimal point, so some specific diagnoses were grouped. While this was not a problem for identification of MS, other groups contained diagnoses which would have been interesting to identify separately, such as clinically isolated syndrome, which is associated with future MS risk,[31] and myelin oligodendrocyte glycoprotein antibody disease, which is a separate disease entity.[32] Lack of a specific diagnosis, may also be because of insufficient follow-up time. However, in the context

For Review Only

of all demyelinating diagnoses, MS is less rare. As diagnostic precision remains an issue, and disease progression is incompletely described, further research should examine evidence of progression of demyelination with information on diagnostic methods, including use of repeated MRI examinations. Many further individuals may have been infected but were not tested by PCR (however, testing during the major period of the study period was extensive, although there was limited testing at the beginning of the pandemic. As we divided the analysis into two periods of infection, this may have helped to identify changes in the PCR positive population, particularly those who were not admitted to hospital. Some demyelinating diseases, particularly MS, are insidious in onset, with development over a long period and so will mostly not have been detected during the limited follow-up time of this study. The SARS-CoV-2 viral variant responsible for the infection could not be identified in our data so calendar period was used as the underlying timescale in the analysis to take aspects of the changing characteristics of the pandemic and vaccine coverage into account. We also divided the period of exposure: up to the end of 2020 and then subsequently. Shared susceptibility to SARS-CoV-2 infection and the outcomes could explain some of the associations. To address this, anyone with a demyelinating diagnosis prior to 2020 was excluded and we adjusted for Charlson Comorbidity Index score to control for underlying health problems: as expected, a higher Charlson Comorbidity Index score was generally positively associated with the outcomes, but the demyelinating outcomes associated with COVID-19 were found most notably among those without comorbidity. Another limitation is that we did not have information on body mass index (BMI), which would have been a potentially useful covariate as obesity is an important risk factor for severe COVID-19. Importantly, the results may have been influenced by surveillance/referral bias, as continued contact with healthcare following hospital admission for COVID-19 may have resulted in diagnosis of demyelinating disease.

For Review Only

We found an increased risk of demyelinating diseases of the CNS among people treated in hospital for COVID-19. A proportion of these associations with COVID-19 may be due to shared susceptibility or surveillance bias. The occurrence of delayed-onset demyelinating diseases should continue to be assessed among those who experienced COVID-19, as some of these diseases can have long asymptomatic and prodromal phases.

Data availability

The data used in this study are deidentified individual level data from Swedish healthcare registers and can be obtained from the respective Swedish public data holders with ethical approval for the research in question, subject to relevant legislation, processes, and data protection. Details of the data can be found here: https://www.gu.se/en/research/scifi-pearl

Funding

This study was funded by grants from *Nyckelfonden*. The SCIFI-PEARL project has basic funding based on grants from the Swedish state under the agreement between the Swedish government and the county councils, the ALF-agreement (*Avtal om Läkarutbildning och Forskning*/Medical Training and Research Agreement) grants ALFGBG-938453, ALFGBG-971130, ALFGBG-978954, and previously from a joint grant from FORTE (*Forskningsrådet för hälsa, arbetsliv och* välfärd/Research Council for Health, Working Life, and Welfare) and FORMAS (*Forskningsrådet för miljö, areella näringar och samhällsbyggande*/Research Council for Environment, Agricultural Sciences and Spatial Planning), grant 2020-02828.

Competing interests

For Review Only

SM has received MS research grants and/or honoraria for advisory boards/lectures from Roche, Novartis, AstraZeneca, Merck, Teva and IQVIA. FN owns AstraZeneca shares. The other authors have no disclosures.

For Review Only

References

1. Ismail, II, Salama S. Association of CNS demyelination and COVID-19 infection: an updated systematic review. *J Neurol* 2022;269(2):541-76. doi: 10.1007/s00415-021-10752-x [published Online First: 2021/08/14]
2. Lotan I, Nishiyama S, Manzano GS, et al. COVID-19 and the risk of CNS demyelinating diseases: A systematic review. *Front Neurol* 2022;13:970383. doi: 10.3389/fneur.2022.970383 [published Online First: 20220920]
3. Arino H, Heartshorne R, Michael BD, et al. Neuroimmune disorders in COVID-19. *J Neurol* 2022;269(6):2827-39. doi: 10.1007/s00415-022-11050-w [published Online First: 2022/03/31]
4. Lanz TV, Brewer RC, Ho PP, et al. Clonally expanded B cells in multiple sclerosis bind EBV EBNA1 and GlialCAM. *Nature* 2022 doi: 10.1038/s41586-022-04432-7 [published Online First: 2022/01/25]
5. Bjornevik K, Cortese M, Healy BC, et al. Longitudinal analysis reveals high prevalence of Epstein-Barr virus associated with multiple sclerosis. *Science* 2022;375(6578):296-301. doi: 10.1126/science.abj8222 [published Online First: 2022/01/14]
6. Xu Y, Hiyoshi A, Smith KA, et al. Association of Infectious Mononucleosis in Childhood and Adolescence With Risk for a Subsequent Multiple Sclerosis Diagnosis Among Siblings. *JAMA Netw Open* 2021;4(10):e2124932. doi: 10.1001/jamanetworkopen.2021.24932 [published Online First: 2021/10/12]
7. Giovannoni G. How long is the presymptomatic phase of multiple sclerosis? *Mult Scler Relat Disord* 2016;7:12-3. doi: 10.1016/j.msard.2016.02.010 [published Online First: 2016/05/31]
8. Xu Y, Smith KA, Hiyoshi A, et al. Hospital-diagnosed infections before age 20 and risk of a subsequent multiple sclerosis diagnosis. *Brain* 2021;144(8):2390-400. doi: 10.1093/brain/awab100 [published Online First: 2021/03/12]
9. Correale J, Fiol M, Gilmore W. The risk of relapses in multiple sclerosis during systemic infections. *Neurology* 2006;67(4):652-9. doi: 10.1212/01.wnl.0000233834.09743.3b [published Online First: 2006/07/28]
10. Nouri MN YE. Neuroinflammatory and Demyelinating Disorders of Childhood. *Clinical Child Neurology* 2020;9:651-77.
11. Nyberg F, Franzen S, Lindh M, et al. Swedish Covid-19 Investigation for Future Insights - A Population Epidemiology Approach Using Register Linkage (SCIFI-PEARL). *Clin Epidemiol* 2021;13:649-59. doi: 10.2147/CLEP.S312742 [published Online First: 2021/08/07]
12. Charlson ME, Pompei P, Ales KL, et al. A new method of classifying prognostic comorbidity in longitudinal studies: development and validation. *J Chronic Dis* 1987;40(5):373-83. doi: 10.1016/0021-9681(87)90171-8 [published Online First: 1987/01/01]
13. Quan H, Li B, Couris CM, et al. Updating and validating the Charlson comorbidity index and score for risk adjustment in hospital discharge abstracts using data from 6 countries. *Am J Epidemiol* 2011;173(6):676-82. doi: 10.1093/aje/kwq433 [published Online First: 2011/02/19]
14. Quan H, Sundararajan V, Halfon P, et al. Coding algorithms for defining comorbidities in ICD-9-CM and ICD-10 administrative data. *Med Care* 2005;43(11):1130-9. doi: 10.1097/01.mlr.0000182534.19832.83 [published Online First: 2005/10/15]

For Review Only

15. Moore L, Ghannam M, Manousakis G. A first presentation of multiple sclerosis with concurrent COVID-19 infection. *eNeurologicalSci* 2021;22:100299. doi: 10.1016/j.ensci.2020.100299 [published Online First: 2020/12/15]
16. Yavari F, Raji S, Moradi F, et al. Demyelinating Changes Alike to Multiple Sclerosis: A Case Report of Rare Manifestations of COVID-19. *Case Rep Neurol Med* 2020;2020:6682251. doi: 10.1155/2020/6682251 [published Online First: 2021/01/12]
17. Smith KA, Hiyoshi A, Burkill S, et al. Hospital diagnosed pneumonia before age 20 years and multiple sclerosis risk. *BMJ Neurol Open* 2020;2(1):e000044. doi: 10.1136/bmjno-2020-000044 [published Online First: 2021/03/09]
18. Loebermann M, Winkelmann A, Hartung HP, et al. Vaccination against infection in patients with multiple sclerosis. *Nat Rev Neurol* 2012;8(3):143-51. doi: 10.1038/nrneurol.2012.8 [published Online First: 2012/01/25]
19. Ryan FJ, Hope CM, Masavuli MG, et al. Long-term perturbation of the peripheral immune system months after SARS-CoV-2 infection. *BMC Med* 2022;20(1):26. doi: 10.1186/s12916-021-02228-6 [published Online First: 2022/01/15]
20. Montgomery S, Hiyoshi A, Burkill S, et al. Concussion in adolescence and risk of multiple sclerosis. *Ann Neurol* 2017;82(4):554-61. doi: 10.1002/ana.25036 [published Online First: 2017/09/05]
21. Montgomery S, Hiyoshi A, Burkill S, et al. Reply to "concussion may not cause multiple sclerosis". *Ann Neurol* 2017;82(4):652-53. doi: 10.1002/ana.25062 [published Online First: 2017/10/05]
22. Rogers JP, Watson CJ, Badenoch J, et al. Neurology and neuropsychiatry of COVID-19: a systematic review and meta-analysis of the early literature reveals frequent CNS manifestations and key emerging narratives. *J Neurol Neurosurg Psychiatry* 2021;92(9):932-41. doi: 10.1136/jnnp-2021-326405 [published Online First: 2021/06/05]
23. Murray RS, Cai GY, Hoel K, et al. Coronavirus infects and causes demyelination in primate central nervous system. *Virology* 1992;188(1):274-84. doi: 10.1016/0042-6822(92)90757-g [published Online First: 1992/05/01]
24. Desforges M, Le Coupanec A, Dubeau P, et al. Human Coronaviruses and Other Respiratory Viruses: Underestimated Opportunistic Pathogens of the Central Nervous System? *Viruses* 2019;12(1) doi: 10.3390/v12010014 [published Online First: 2019/12/22]
25. Cao X. COVID-19: immunopathology and its implications for therapy. *Nat Rev Immunol* 2020;20(5):269-70. doi: 10.1038/s41577-020-0308-3 [published Online First: 2020/04/11]
26. Kostic M, Dzopalic T, Zivanovic S, et al. IL-17 and glutamate excitotoxicity in the pathogenesis of multiple sclerosis. *Scand J Immunol* 2014;79(3):181-6. doi: 10.1111/sji.12147 [published Online First: 2014/01/05]
27. Odoardi F, Sie C, Streyl K, et al. T cells become licensed in the lung to enter the central nervous system. *Nature* 2012;488(7413):675-9. doi: 10.1038/nature11337 [published Online First: 2012/08/24]
28. Tzartos JS, Friese MA, Craner MJ, et al. Interleukin-17 production in central nervous system-infiltrating T cells and glial cells is associated with active disease in multiple sclerosis. *Am J Pathol* 2008;172(1):146-55. doi: 10.2353/ajpath.2008.070690 [published Online First: 2007/12/25]

For Review Only

29. Cepok S, Zhou D, Srivastava R, et al. Identification of Epstein-Barr virus proteins as putative targets of the immune response in multiple sclerosis. *J Clin Invest* 2005;115(5):1352-60. doi: 10.1172/JCI23661 [published Online First: 2005/04/21]
30. Lindsey JW. Antibodies to the Epstein-Barr virus proteins BFRF3 and BRRF2 cross-react with human proteins. *J Neuroimmunol* 2017;310:131-34. doi: 10.1016/j.jneuroim.2017.07.013 [published Online First: 2017/08/06]
31. Filippi M, Preziosa P, Meani A, et al. Prediction of a multiple sclerosis diagnosis in patients with clinically isolated syndrome using the 2016 MAGNIMS and 2010 McDonald criteria: a retrospective study. *Lancet Neurol* 2018;17(2):133-42. doi: 10.1016/S1474-4422(17)30469-6 [published Online First: 20171221]
32. Banwell B, Bennett JL, Marignier R, et al. Diagnosis of myelin oligodendrocyte glycoprotein antibody-associated disease: International MOGAD Panel proposed criteria. *Lancet Neurol* 2023;22(3):268-82. doi: 10.1016/S1474-4422(22)00431-8 [published Online First: 20230124]

For Review Only

For Review Only

Table 1. Baseline characteristics of the study population (N=9 981 915) aged between 3 and 100 years in January 2020, by subsequent SARS-CoV-2 infection status by 30th November 2022

	No diagnosed infection Total n=7,498,492 N (%)	Positive SARS-CoV-2 test only Total n=2,371,402 N (%)	Hospital admission due to SARS-CoV-2 Total n=112,021) N (%)
**Age** (years)			
3-10	716 034 (9.6)	205 879 (8.7)	794 (0.7)
11-20	851 939 (11.4)	323 550 (13.6)	1718 (1.5)
21-30	926 137 (12.4)	410 063 (17.3)	5174 (4.6)
31-40	908 194 (12.1)	444 038 (18.7)	7343 (6.6)
41-50	868 125 (11.58)	421 281 (17.8)	10 194 (9.1)
51-60	960 263 (12.8)	317 894 (13.4)	15 874 (14.2)
61-70	958 268 (12.8)	130 613 (5.5)	18 617 (16.6)
71-80	882 771 (11.8)	63 011 (2.7)	26 547 (23.7)
81-90	357 146 (4.8)	41 226 (1.7)	20 809 (18.6)
91-100	69 615 (0.9)	13 847 (0.6)	4951 (4.4)
**Sex**			
Male	3 856 628 (51.4)	1 100 319 (46.4)	62 231 (55.6)
Female	3 641 864 (48.6)	1 271 083 (53.6)	49 790 (44.5)
**Charlson Comorbidity Index**			
0	6 561 231 (87.5)	2 166 234 (91.4)	62 858 (56.1)
1	358 769 (4.8)	101 845 (4.3)	12 966 (11.6)
2	372 423 (5.0)	71 989 (3.0)	16 980 (15.2)
3 or more	206 069 (2.8)	31 334 (1.3)	19 217 (17.2)
**Region of Sweden**			
North	668 495 (8.9)	180 217 (7.6)	8151 (7.3)
South	1 335 440 (17.8)	436 487 (18.4)	16 198 (14.5)
Stockholm	1 723 729 (23.0)	554 305 (23.4)	31 734 (28.3)
South East	783 644 (10.5)	230 028 (9.7)	11 224 (10.0)
Uppsala-Örebro	1 500 674 (20.0)	502 513 (21.2)	20 488 (18.3)
West	1 350 693 (18.0)	461 271 (19.5)	17 054 (15.2)
Other	135 817 (1.8)	6 581 (0.3)	7 172 (6.4)
**Region of origin**			
Africa	180 238 (2.4)	48 191 (2.0)	3 044 (2.7)
Asia	569 864 (7.6)	200 446 (8.5)	12 766 (11.4)
European Union excluding Nordic countries	297 723 (4.0)	77 320 (3.3)	4 408 (3.9)
Europe excluding European Union and Nordic countries	185 408 (2.5)	76 236 (3.2)	5 776 (5.2)
North America	31 692 (0.4)	9 259 (0.4)	393 (0.4)
Nordic countries excluding Sweden	189 343 (2.5)	36 599 (1.5)	4980 (4.5)
Oceania	4 998 (0.1)	1353 (0.1)	19 (0.0)
Former Soviet Union	4 199 (0.1)	1066 (0.0)	121 (0.1)
Sweden	5 981 516 (79.8)	1 899 892 (80.1)	79 093 (70.6)
South America	52 119 (0.7)	20 763 (0.9)	1408 (1.3)
Other	1 392 (0.0)	277 (0.0)	13 (0.0)
**Demyelinating disease diagnoses prior to 2020**			
No	7 483 154 (99.8)	2 366 117 (99.8)	111 106 (99.2)
Yes	15 338 (0.2)	5285 (0.2)	915 (0.8)

For Review Only

Table 2. Hazard ratios (HR) with 95% confidence intervals (CI) for non-MS demyelinating diseases associated with SARS-CoV-2 status

	N non-MS demyelinating diseases/N total	Rate† (95% CI)	Unadjusted HR (95% CI)	P	Adjusted** HR (95% CI)	P
**Total**	1124/9 959 818	4.0 (3.7 to 4.2)				
**SARS-CoV-2***						
Not diagnosed	960/9 959 776	3.8 (3.6 to 4.0)	Reference		Reference	
Positive test only	152/2 386 953	5.1 (4.3 to 6.0)	1.30 (1.09 to1.56)	0.004	1.06 (0.88 to1.27)	0.541
Hospital admission	12/107 282	9.0 (5.1 to 15.9)	2.33 (1.32 to 4.12)	0.004	2.36 (1.33 to 4.19)	0.003
**Sex**						
Male	386/5 012 336	2.7 (2.4 to 3.0)	Reference		Reference	
Female	738/4 947 482	5.2 (4.9 to 5.6)	1.94 (1.71 to 2.19)	<0.001	1.99 (1.76 to 2.25)	<0.001
**Charlson Comorbidity Index**						
0	969/8 772 348	3.8 (3.6 to 4.1)	Reference		Reference	
1	59/472 485	4.4 (3.4 to 5.7)	1.14 (0.88 to 1.49)	0.318	1.50 (1.15 to 1.95)	0.003
2	71/459 256	5.7 (4.5 to 7.2)	1.49 (1.17 to 1.90)	0.001	2.24 (1.75 to 2.88)	<0.001
3 or more	25/255 729	4.3 (2.9 to 6.3)	1.12 (0.75 to 1.67)	0.569	1.88 (1.25 to 2.83)	0.002

*SARS-CoV-2 was modelled as a time-varying exposure, hence the total sum of number of observations (individuals) contributing time at risk in each category of SARS-CoV-2 exposure is greater than the total number of individuals.
** Adjusted for birth year (1920-1940, 1941-1960, 1961-1980, 1981-2000, 2001-2016), sex (male, female), regions of Sweden (North, South, Stockholm, South East, Uppsala-Örebro, West, other), region of origin (Africa, Asia, European Union excluding Nordic countries, Europe excluding European Union and Nordic countries, North America, Nordic countries excluding Sweden, Oceania, former Soviet Union, Sweden, South America, other), and Charlson comorbidity index.
†Per 100,000 person-years.

For Review Only

Table 3. Demyelinating diseases (excluding MS), by SARS-CoV-2 infection status by November 2022

ICD-10		Not diagnosed, n=969	Positive SARS-CoV-2 test, only n=158	Hospital admission, n=12
**G36**	**Other acute disseminated demyelination**			
G36.0	Neuromyelitis optica [Devic]	89 (8.8%)	11 (6.7%)	2 (16.7%)
G36.1	Acute and subacute haemorrhagic leukoencephalitis [Hurst]	-	1 (0.6%)	-
G36.8	Clinically isolated syndrome and other specified acute disseminated demyelination	9 (0.9%)	4 (2.4%)	1 (8.3%)
G36.9	Acute disseminated demyelination, unspecified	23 (2.3%)	3 (1.8%)	-
**G37**	**Other demyelinating diseases of the central nervous system**			
G37.0	Diffuse sclerosis	4 (0.4%)	1 (0.6%)	-
G37.1	Central demyelination of corpus callosum	4 (0.4%)	-	1 (8.3%)
G37.2	Central pontine myelinolysis	38 (3.8%)	1 (0.6%)	-
G37.3	Acute transverse myelitis in demyelinating disease of central nervous system	32 (3.2%)	2 (1.2%)	-
G37.4	Subacute necrotizing myelitis	2 (0.2%)	1 (0.6%)	-
G37.5	Concentric sclerosis [Baló]	1 (0.1%)	-	-
G37.8	Myelin oligodendrocyte glycoprotein antibody disease and other specified demyelinating diseases of central nervous system	68 (6.7%)	12 (7.3%)	1 (8.3%)
G37.9	Demyelinating disease of central nervous system, unspecified	740 (73.3%)	129 (78.2%)	7 (58.3%)

Some individuals had more than one demyelinating disease diagnosis.
The percentages indicate proportions only among those with a demyelinating disease and cannot be used to estimate relative risk from this table alone.

For Review Only

Table 4. Hazard ratios (HR) with 95% confidence intervals (CI) for multiple sclerosis associated with SARS-CoV-2 status

	N multiple sclerosis/ N total	Rate† (95% CI)	Unadjusted HR (95% CI)	P	Adjusted** HR (95% CI)	P
**Total**	2787/9 959 816	9.8 (9.4 to 10.2)				
**SARS-CoV-2***						
Not diagnosed	2403/9 959 774	9.5 (9.1 to 9.9)	Reference		Reference	
Positive test only	356/2 386 629	11.9 (11.7 to 13.2)	1.30 (1.16 to 1.47)	<0.001	1.08 (0.96 to1.22)	0.200
Hospital admission	28/107 246	21.1 (14.6 to 30.5)	2.28 (1.57 to 3.31)	<0.001	2.48 (1.70 to 3.61)	<0.001
**Sex**						
Male	926/5 012 335	6.5 (6.1 to 6.9)	Reference		Reference	
Female	1861/4 947 481	13.2 (12.6 to 13.8)	2.03 (1.88 to 2.20)	<0.001	2.08 (1.92 to 2.25)	<0.001
**Charlson Comorbidity Index**						
0	2470/8 772 346	9.8 (9.4 to 10.2)	Reference		Reference	
1	120/472 485	8.9 (7.5 to 10.7)	0.91 (0.76 to 1.10)	0.323	1.14 (0.95 to 1.37)	0.157
2	149/459 256	12.0 (10.2 to 14.1)	1.23 (1.04 to 1.45)	0.015	1.60 (1.35 to 1.90)	<0.001
3 or more	48/255 729	8.2 (6.2 to 10.9)	0.84 (0.63 to 1.12)	0.227	1.15 (0.86 to 1.55)	0.342

*SARS-CoV-2 was modelled as a time-varying exposure, hence the total number of observations in each category of SARS-CoV-2 exposure is greater than the total number of individuals.

** adjusted for birth year (1920-1940, 1941-1960, 1961-1980, 1981-2000, 2001-2016), sex (male, female), regions of Sweden (North, South, Stockholm, South East, Uppsala-Örebro, West, other), region of origin (Africa, Asia, European Union excluding Nordic countries, Europe excluding European Union and Nordic countries, North America, Nordic countries excluding Sweden, Oceania, former Soviet Union, Sweden, South America, other), and Charlson Comorbidity Index.

†Per 100,000 person-years.

For Review Only

Table 5. Hazard ratios (HR) with 95% confidence intervals (CI) for non-MS demyelinating disease and multiple sclerosis outcomes associated with SARS-CoV-2 status before and from 1st January, 2021.

SARS-CoV-2*	N non-MS demyelinating diseases/N total	Adjusted** HR (95% CI)	P	N multiple sclerosis/ N total	Adjusted** HR (95% CI)	P
**Total**	1124/9 959 818			2787/9 959 816		
Not diagnosed	960/9 959 776	Reference		2403/9 959 774	Reference	
Positive test only before 01/01/2021	42/438 017	0.96 (0.71 to 1.32)	0.823	112/437 987	1.10 (0.91 to 1.34)	0.319
Positive test only from 01/01/2021	110/1 948 936	1.10 (0.89 to 1.36)	0.366	244/1 948 642	1.07 (0.93 to 1.23)	0.332
Hospital admission before 01/01/2021	7/39 576	2.60 (1.23 to 5.49)	0.012	11/39 564	1.85 (1.02 to 3.36)	0.042
Hospital admission from 01/01/2021	5/67 706	2.09 (0.86 to 5.05)	0.102	17/67 682	3.18 (1.97 to 5.13)	<0.001

*SARS-CoV-2 was modelled as a time-varying exposure, hence the total number of observations in each category of SARS-CoV-2 exposure is greater than the total number of individuals.
** Adjusted for birth year (1920-1940, 1941-1960, 1961-1980, 1981-2000, 2001-2016), sex (male, female), regions of Sweden (North, South, Stockholm, South East, Uppsala-Örebro, West, other), region of origin (Africa, Asia, European Union excluding Nordic countries, Europe excluding European Union and Nordic countries, North America, Nordic countries excluding Sweden, Oceania, former Soviet Union, Sweden, South America, other), and Charlson Comorbidity Index.

For Review Only

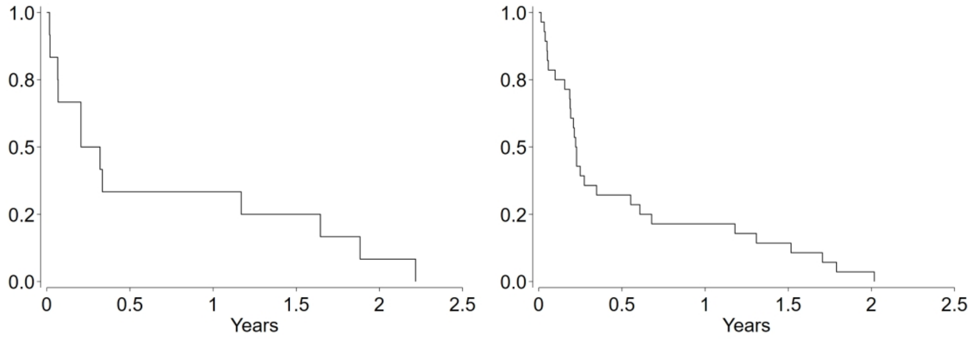

Figure 1 Timing of demyelinating disease diagnoses following hospital admission for COVID-19. The Kaplan-Meier curves show the duration from hospital admission for COVID-19, modelled as a time-dependent variable, to (A, left) diagnosis of a non-MS demyelinating disease and (B, right) diagnosis of MS.

867x318mm (72 x 72 DPI)
