# Supplementary material for: SARS-CoV-2 infection and risk of subsequent demyelinating diseases: national register–based cohort study
Source: Brain Commun. 2024 Nov 29;6(6):fcae406. doi: 10.1093/braincomms/fcae406 (PMC11629974; doi:10.1093/braincomms/fcae406)
Supplement: fcae406_Supplementary_Data [file fcae406_supplementary_data.zip › Supplementary Materials.pdf]

```
*-------------------------------------------------------------------*
* Stata code used for the analysis
*-------------------------------------------------------------------*
* The analysis for non-MS demyelinating disease and MS were
conducted by changing the fu_end and f().
* fu_end: variable indicating the date of the end of follow-up
* outcome: variable indicating failure(=1, otherwise 0)

* Declare data for survival-time (time scale: time-since-entry)
gen entry = mdy(1,1,2020)
format entry %td
stset fu_end, f(outcome) origin(entry) entry(entry) id(ID)
scale(365.25)

*Time-varying positive test exposure
gen c19_d1_index_copy=c19_d1_index
replace c19_d1_index_copy=mdy(1,1,2030) if c19_d1_index_copy==.
format c19_d1_index_copy %td
stsplit tvc19_d1, at(0) after(time=c19_d1_index_copy)
replace tvc19_d1=tvc19_d1+1
label define c19 0 "0.Unexposed" 1 "1.Exposed", replace
label value tvc19_d1 c19

*Time-varying hospital admissions exposure
gen c19_h2_index_copy=c19_h2_index
replace c19_h2_index_copy=mdy(1,1,2030) if c19_h2_index_copy==.
format c19_h2_index_copy %td
stsplit tvc19_h2, at(0) after(time=c19_h2_index_copy)
replace tvc19_h2=tvc19_h2+1
label value tvc19_h2 c19

*Combining 2 exposure variables into one
gen tvc19_2cat=tvc19_d1+tvc19_h2
label define tvcovid 0 "0.Unexposed" 1 "1.+ve test" 2 "2.hosp/ICU",
replace
label value tvc19_2cat tvcovid
tab tvc19_2cat _d if _st==1,m

* Tab2/4 rates
strate, per(100000)
strate tvc19_2cat, per(100000)
strate gender, per(100000)
strate cci_4cat, per(100000)

* Tab2/4 HRs
stcox i.tvc19_2cat, base
stcox i.gender, base
stcox i.cci_4cat, base
stcox i.tvc19_2cat i.gender i.cci_4cat ib3.birthyearcat_5
ib3.healthreg_no_m ib9.country, base

* Tab5
label define tvc19_4cat 0 "0.Unexposed" 1 "+ve <2021" 2 "2.+ve
>=2021" 3 "3.hosp/ICU <2021" 4 "4.hosp/ICU >=2021", replace
gen tvc19_4cat=tvc19_2cat
recode tvc19_4cat 2=4 if tvc19_4cat==2 & _t0>=1 & _t0<3 & _st==1
```

```
recode tvc19_4cat 2=3 if tvc19_4cat==2 & _t0>=0 & _t0<1 & _st==1
recode tvc19_4cat 1=2 if tvc19_4cat==1 & _t0>=1 & _t0<3 & _st==1
label value tvc19_4cat tvc19_4cat

tab tvc19_4cat tvc19_2cat if _st==1,m
tab tvc19_4cat _d if _st==1,m

stcox i.tvc19_4cat i.gender i.cci_4cat ib3.birthyearcat_5
ib3.healthreg_no_m ib9.country, base
```
