# Supplementary material for: SARS-CoV-2 infection and risk of subsequent demyelinating diseases: national register–based cohort study
Source: Brain Commun. 2024 Nov 29;6(6):fcae406. doi: 10.1093/braincomms/fcae406 (PMC11629974; doi:10.1093/braincomms/fcae406)
Supplement: fcae406_Supplementary_Data [file Original_submission_decision_letter.pdf › Original_submission_decision_letter.pdf]

Original Decision Letter

25th March 2024

Dear Dr. Montgomery,

Thank you for your recent submission BRAINCOM-2024-038 entitled "SARS-CoV-2 infection and risk of subsequent demyelinating diseases: national register-based cohort study" to Brain Communications.

Your manuscript has been reviewed, and while the reviewers and editorial team appreciate many aspects of the paper, they raise some concerns that you will need to address in order for the paper to be considered for publication.

If you can address these concerns, I invite you to submit a revised manuscript and a response to each of the reviewers' comments. The reviewer(s)' comments are included at the bottom of this message.

In addition to the reviewers' comments, please also address our Scientific Editor's comments added below to comply with our requirements for presentation of scientific data at Brain Communications.

Please format your revised manuscript according to our Instructions to Authors
https://academic.oup.com/braincomms/pages/General_Instructions
A summary of key points is attached to help with this. Please note that there can be substantial delays to publication if you don't resubmit in line with our requirements.

To revise your manuscript, click the link below or log into https://mc.manuscriptcentral.com/braincom and enter your Author Centre, where you will find your manuscript title listed under "Manuscripts with Decisions." Under "Actions," click on "Create a Revision." Your manuscript number will be appended to denote a revision.

*** PLEASE NOTE: This is a two-step process. After clicking on the link, you will be directed to a webpage to confirm. ***

https://mc.manuscriptcentral.com/braincom?URL_MASK=1f3f8df0a6004692b20f6c772bec7c5f

Please upload a revised manuscript with changes tracked with track changes or in

coloured text.

When submitting your revised manuscript, you will be asked to respond to the reviewer(s) comments. Please be as specific as possible in your response to all of the comments.

The editorial team at Brain Communications aims to increase transparency in scientific publishing and to demonstrate the value that peer review brings to the scientific process. Please therefore consider whether you are willing to have reviewer(s)' comments on your paper and your response published as supplementary data with the paper if it is accepted. This is entirely optional and will only be done if both the authors and reviewers agree. While submitting your revision there will be an opportunity to review or update the answer you gave previously, if necessary.

IMPORTANT: Your original files are available to you when you upload your revised manuscript. Please delete any redundant files before completing the submission.

Because we are trying to facilitate timely publication of manuscripts submitted to Brain Communications, we would appreciate your revised manuscript as soon as possible. If it is not possible for you to resubmit within 3 months, the option to submit a revision will expire, and we would then consider your revised paper as a new submission. However, if you need a little more time, please get in touch and we can extend this deadline for you.

Once again, thank you for submitting your manuscript to Brain Communications and I look forward to receiving your revision.

Best wishes

Dr. Josef Priller
Associate Editor, Brain Communications

Professor Tara Spires-Jones
Founding Editor, Brain Communications

Reviewer(s)' Comments to Author:

Reviewer: 1

Comments to the Author
Montgomery et al. report the results of a national register-based cohort study evaluating the association between SARS-CoV-2 and the occurrence of subsequent demyelinating diseases of the CNS, as well as of EBV infection. The authors show that a severe SARS-CoV-2 infection (defined as an infection requiring hospitalization) is associated with a significant risk of developing non-MS demyelinating disease, MS, and infectious mononucleosis (IM) over a surveillance period of about three years.
My two main concerns are that the definition of non-MS demyelinating disease is vague and includes some disease entities that are rarely used nowadays. At the same time, it is unclear whether other clinical entities, for example, clinically isolated syndrome (CIS) and myelin oligodendrocyte glycoprotein antibody-associated disease (MOGAD, which sometimes follows an infection), were included. Also, the inclusion of EBV infection as a primary outcome of interest for this study is questionable. While the authors refer to some recent publications that report a significant association between prior EBV infection and the occurrence of MS, I am not sure I would call it an established risk factor. And even if it is, the current literature suggests that a remote EBV infection may be associated with the subsequent occurrence of MS many years after the infection. Here, the authors look at the association between acute SARS-CoV-2 infections and MS/other non-MS demyelinating diseases during a relatively short period. Adding EBV infection as another outcome in this context does not make sense to me, especially when none of the demyelinating cases occurred after EBV infection. Why focus only on EBV and no other risk factors? I would personally suggest focusing on demyelinating diseases only.

Below, please see some more detailed comments:

Introduction:
- Not all non-MS demyelinating diagnoses are considered risk factors for MS. Please be more specific. Other than clinically isolated syndrome (CIS, which is not listed among the possible diagnoses), what clinical entities are you referring to?
- At least one more systematic review concerning the association between SARS-CoV-2 infection and subsequent demyelinating CNS disease has been published (PMID: 36203986). Please add it as well.
- When discussing “other demyelinating diagnoses,” please specify which diagnoses you are referring to.
Methods:
- If the Swedish database was established to investigate COVID-19, why was data

available from 2015?
- The authors applied the Charlson Comorbidity Index, commonly used to predict survival, as a general index of associated comorbidities. I assume it was applied to evaluate whether people with comorbidities are at higher risk for severe COVID-19. However, it does not include obesity, which is considered a risk factor for severe COVID-19.
- Under "country of birth," there are mostly geographical areas, not specific countries.
Outcomes:
- The types of non-MS demyelinating diagnoses should be specified more clearly. Looking at Table 3, many diagnoses are somewhat ambiguous and not used in common practice, and MOGAD is not included. If that is the case, the authors should mention it and acknowledge it as a significant limitation.
Statistical analysis:
- I agree that a confidence interval not including 1.0 signifies statistical significance. However, the more commonly used statistical marker for significance is the p-value. As the p values are reported in the tables, it would make more sense to refer to it in the text as well.
Results:
- The sentence: Those admitted to hospital were more likely to have preexisting poorer health, as indicated by the Charlson Comorbidity Index and demyelinating disease prior to 2020" is confusing. Do you mean that a prior diagnosis of demyelinating disease is a risk factor for severe COVID-19? Then, in the following sentence, you say that all prior demyelinating diseases were excluded. Please clarify/rephrase.
- Please add p-values.
- What do you mean by "the outcomes were rare" (page #10)?
- The list of non-MS demyelinating diseases other than neuromyelitis optica is vague and unclear (page #10).
- The median age at MS diagnosis in patients with prior SARS-CoV-2 infection was 56.7 years, which is rather old compared to the usual age of MS diagnosis. It would be interesting to know whether these newly diagnosed MS cases were of the relapsing or the progressive form (which tends to occur in older age).
- The paragraph describing the stratified analysis is hard to read. I am also not sure how informative it is.

Discussion:
- In my opinion, the Discussion is too long and hard to follow. I would try to focus on the main findings and explain them, possibly leaving out the part regarding EBV infection (which is interesting but does not serve the scope of this paper and is somewhat hypothetical).
- It is unclear which non-MS demyelinating disease the authors refer to as a risk factor for subsequent MS (page #14, lines # 10-12). For example, NMO is not a risk factor for

MS.
- In the limitations, please mention that MOGAD was not included as a diagnosis.

General comment: please check the text for grammatical errors and typos (i.e., unnecessary “(“marks, etc.).

Reviewer: 2

Comments to the Author
In this study the authors sought to understand the potential relationship between COVID infection and subsequent demyelinating disease. This is an area of great interest, as in the context of a novel pathogen, subsequent long term inflammatory conditions are a concern. As the authors imply, there is already strong associative evidence linking MS with EBV, and so links with COVID and EBV reactivation are a natural area of interest, particularly where high quality records and linkage data is available.

It is interesting that the signal is seen around hospital admissions (taken as a proxy for infection severity, which is appropriate), but not with PCR positive cases. Was PCR only available in symptomatic cases? Data suggests that a substantial proportion of COVID infection is asymptomatic, even in the pre-vaccine era. Was this accounted for at all in the analysis?

I do have some further concerns linked to this.

Firstly, as the authors state, the assumed lag time is short between COVID and subsequent MS. Given current understanding around prodromal MS, and lag between radiological and clinical onset in many, there may be insufficient time for this study to really unpick this relationship. Further, the relationship that is seen may be due to (early) ascertainment bias, with those treated in hospital more likely to undergo neuroimaging, and so be diagnosed earlier than they might otherwise have been. This would potentially be supported by the lack of signal with non-hospitalised patients. I think this point requires further discussion.

Second, the number with inflammatory CNS disease following hospital admission with COVID is small - 12 in total, 7 of whom have unspecified neuroinflammation. I think that the text of the paper needs to be clearer regarding the small number and non-specific diagnoses that the positive conclusions are based on.

I found the data around infectious mononucleosis interesting, however this felt a little

disjointed from the inflammatory neurological diseases. There are challenges with healthcare-records reported IM, with relatively poor sensitivity and specificity overall. I am concerned that the symptoms of IM and "long COVID" may have substantial overlap and so recording bias may have driven this finding. Was IM evaluated using monospot or other laboratory test in any/some/all cases? This needs to be considered and discussed.

More minor comments:
Whilst date is controlled for I could not see that vaccination status was considered? Is this data available and should it be included in the analysis?
I think the abstract should be toned down in terms of findings, as it was not immediately clear that the positive findings related to hospital admissions only (and the small numbers) rather than COVID infection overall.
Even though the timescale of the study is short, I wonder if survival curves showing time from infection to diagnosis of neuroinflammatory disease might help the reader.

Comments from the Scientific Editorial team:

-Please add an Abbreviated Summary (up to 50 words) of your paper that captures the main purpose and conclusions of the work. The summary should state the main results written in the third person ('[1st author surname] et al. report that ...') and conclusion. This summary will be used in the contents list online and should not contain any abbreviations other than those on the accepted abbreviations list and accepted gene/protein names, making it accessible to the non-specialist. Please upload it as a Word document. Allowed abbreviations are: AIDS; ANOVA; AMPA; ATP; A,T,C,G; CNS; CSF; CT; DNA; ECG; EEG; EMG; GABA; HIV; MRI; NMDA; PET; PCR; RNA.

-Please provide a graphical abstract. A graphical abstract is a visual representation of the central finding or methodology of the paper. It will be displayed in the table of contents. Authors should strive to make the graphical abstract informative, interesting, visually appealing, and straightforward (no caption needed, no abbreviations except gene names). The technical requirements for the graphical abstracts are:
- Size: 120 x 120 mm (300-600dpi) or 3000 x 3000 pixels
- Font: Arial 12-16 pt
- File type: tiff or PDF
- Aspect ratio: 1:1 preferred

-Thank you for providing a data availability statement. Please also provide the codes generated and used in this work within the supplementary material or through a repository, of which the link has to be made available within the data availability

statement.

-Tables 4, 5, 6: These tables are likely to exceed one journal page. Large tables that are more than one page in length, particularly in landscape orientation, are difficult to read and are not usually allowed; such tables should be uploaded as Supplementary material. Supplementary tables containing large amounts of text can be summarized in a smaller table for publication in the article if appropriate. Alternatively, you could consider splitting a large table into smaller tables. Please use the attached template to format your tables, and to check whether they are likely to exceed one typeset journal page.

-As a reminder at this revision stage, if you plan to edit the current figures and tables, or to make new figures and tables, please ensure that these items fit a typeset journal page (A4), or are uploaded as supplementary material.
