# Supplementary material for: SARS-CoV-2 infection and risk of subsequent demyelinating diseases: national register–based cohort study
Source: Brain Commun. 2024 Nov 29;6(6):fcae406. doi: 10.1093/braincomms/fcae406 (PMC11629974; doi:10.1093/braincomms/fcae406)
Supplement: fcae406_Supplementary_Data [file Original_submission_Response_to_reviewers_comments.pdf › Original_submission_Response_to_reviewers_comments.pdf]

Original Submission Response to Reviewers

Dear Editor,

Please consider our revised paper “SARS-CoV-2 infection and risk of subsequent demyelinating diseases: national register-based cohort study” for publication in Brain Communications as an original article.

We have addressed each comment made by the referees and editor, resulting in notable changes and, hopefully, improvements to the manuscript. We have provided our point-by-point responses.

We have added a figure as requested, divided into panels A and B. We could not add the A and B to label the panels in the correct place in the tif file, but perhaps this can be done at the production stage if the paper is accepted. For clarity in the version for referees, we have added ‘left’ and ‘right’ in the figure description, and again this can be altered for production.

Thank you for reconsidering our paper.

Yours faithfully

Scott Montgomery

Please find below our point-by-point responses to the reviewers' comments.

Reviewer(s)' Comments to Author:

Reviewer: 1

Comments to the Author
Montgomery et al. report the results of a national register-based cohort study evaluating the association between SARS-CoV-2 and the occurrence of subsequent demyelinating diseases of the CNS, as well as of EBV infection. The authors show that a severe SARS-CoV-2 infection (defined as an infection requiring hospitalization) is associated with a significant risk of developing non-MS demyelinating disease, MS, and infectious mononucleosis (IM) over a surveillance period of about three years.
My two main concerns are that the definition of non-MS demyelinating disease is vague and includes some disease entities that are rarely used nowadays. At the same time, it is unclear whether other clinical entities, for example, clinically isolated syndrome (CIS) and myelin oligodendrocyte glycoprotein antibody-associated disease (MOGAD, which sometimes follows an infection), were included. Also, the inclusion of EBV infection as a primary outcome of interest for this study is questionable. While the authors refer to some recent publications that report a significant association between prior EBV infection and the occurrence of MS, I am not sure I would call it an established risk factor. And even if it is, the current literature suggests that a remote EBV infection may be associated with the subsequent occurrence of MS many years after the infection. Here, the authors look at the association between acute SARS-CoV-2 infections and MS/other non-MS demyelinating diseases during a relatively short period.

1. Adding EBV infection as another outcome in this context does not make sense to me, especially when none of the demyelinating cases occurred after EBV infection.

Authors' reply
We have removed the sections on EBV-IM as an outcome from the manuscript. We only mention EBV-IM as a potential mediating factor (for which we found no evidence).

2. Why focus only on EBV and no other risk factors? I would personally suggest focusing on demyelinating diseases only.'

Authors' reply
We have reformulated the manuscript throughout to focus only on demyelinating disease risk.

<u>Referee</u>
Below, please see some more detailed comments:

Introduction:

3.- Not all non-MS demyelinating diagnoses are considered risk factors for MS. Please be more specific. Other than clinically isolated syndrome (CIS, which is not listed among the possible diagnoses), what clinical entities are you referring to?

Authors' reply
We have revised the introduction and discussion, so the focus is on demyelinating disease, rather than considering risk factors for MS (especially now IM has been removed). The issue of combined diagnostic categories is tackled as follows. The specificity of demyelinating diseases was limited by the ICD codes available: while this is not an issue for MS and some of the other categories, we have added more detail to indicate the important diagnoses included in some categories. G36.8 is now described as clinically isolated syndrome and other specified acute demyelination. G37.8 is labelled as myelin oligodendrocyte glycoprotein antibody disease and other specified diseases of the central nervous system. We would have required the last digit (G37.81) to be certain of a MOGAD diagnosis, even though this is probably most likely in G37.8. The additional digit is not available in the Swedish version of ICD-10. This is clarified in the results and discussion sections. This limitation is now also emphasised in the discussion as follows.
"The Swedish ICD codes for the outcome diagnoses were limited to one digit after the decimal point, so some specific diagnoses were grouped. While this was not a problem for identification of MS, other groups contained diagnoses which would have been interesting to identify separately, such as clinically isolated syndrome, which is associated with future MS risk,[31] and myelin oligodendrocyte glycoprotein antibody disease, which is a separate disease entity.[32] "

Referee
4.- At least one more systematic review concerning the association between SARS-CoV-2 infection and subsequent demyelinating CNS disease has been published (PMID: 36203986). Please add it as well.

Authors' response

The reference has been added to the introduction and discussion, with a comment in the discussion section.
"Another systematic review concluded that CNS demyelinating events associated with SARS-CoV-2 infections occurred at a low rate, consistent with the low rates observed in our study.[2]"

5. - When discussing "other demyelinating diagnoses," please specify which diagnoses you are referring to.

Authors' response

The specificity of demyelinating diseases was limited by the Swedish ICD codes available: while this is not an issue for MS and some of the other categories, we have added more detail to indicate the important diagnoses included in some categories. G36.8 is now described as clinically isolated syndrome and other specified acute demyelination. G37.8 is labelled as myelin oligodendrocyte glycoprotein antibody disease and other specified diseases of the central nervous system. We would have required an additional digit (G37.81) to be certain of a

MOGAD diagnosis, even though this is probably most likely in G37.8. This is clarified in the results and discussion sections. This limitation is now also emphasised in the discussion as follows.
"The Swedish ICD codes for the outcome diagnoses were limited to one digit after the decimal point, so some specific diagnoses were grouped. While this was not a problem for identification of MS, other groups contained diagnoses which would have been interesting to identify separately, such as clinically isolated syndrome, which is associated with future MS risk,[31] and myelin oligodendrocyte glycoprotein antibody disease, which is a separate disease entity.[32] "

Methods:
6. - If the Swedish database was established to investigate COVID-19, why was data available from 2015?

Authors' response
Data prior to the pandemic were included to identify pre-existing risk factors and other characteristics. These data were from pre-existing national registers. This information has been added to the methods section as follows:
"Most data for the study came from national registers and were available from 1st of January 2015 to 30th November 2022 to identify pre-existing risk factors and temporal trends pre-dating the pandemic."

7. - The authors applied the Charlson Comorbidity Index, commonly used to predict survival, as a general index of associated comorbidities. I assume it was applied to evaluate whether people with comorbidities are at higher risk for severe COVID-19. However, it does not include obesity, which is considered a risk factor for severe COVID-19.

Authors' response
Yes, the Charlson Comorbidity Index was included to tackle potential confounding, as it is associated with COVID-19 severity and possibly risk of some demyelinating diseases. In general, it is not possible to obtain BMI for most of the general population using Swedish registers. This has been added to the limitations section of the discussion. We have added the following to the discussion.
"Another limitation is that we did not have information on body mass index (BMI), which would have been a potentially useful covariate as obesity is an important risk factor for severe COVID-19."

8. - Under "country of birth," there are mostly geographical areas, not specific countries.

Authors' response

This term has been altered to 'region' of birth throughout the manuscript and tables.

Outcomes:
9. - The types of non-MS demyelinating diagnoses should be specified more clearly. Looking at Table 3, many diagnoses are somewhat ambiguous and not used in common practice, and MOGAD is not included. If that is the case, the authors should mention it and acknowledge it as a significant limitation.

Authors' response
All available demyelinating diseases identified by Swedish ICD-10 codes are included, although the level of precision of these codes do not allow us to identify some more specific diagnoses. MOGAD is G37.81 but the precision of our Swedish ICD codes can only identify G37.8 (this diagnosis is associated with Covid-19 in our results). There are only two more detailed G37.8 codes: G37.81 Myelin oligodendrocyte glycoprotein antibody disease; and G37.89 which is 'other specified demyelinating diseases of the central nervous system'. While it most likely that the outcome we identify is MOGAD, we do not want to overinterpret the results. The important limitation of lack of coding specificity is now added to the limitation section of the discussion.

We have altered the disease categories in the tables and added the following to the discussion:
"The Swedish ICD codes for the outcome diagnoses were limited to one digit after the decimal point, so some specific diagnoses were grouped. While this was not a problem for identification of MS, other groups contained diagnoses which would have been interesting to identify separately, such as clinically isolated syndrome, which is associated with future MS risk,[31] and myelin oligodendrocyte glycoprotein antibody disease, which is a separate disease entity.[32]"

Statistical analysis:
10. - I agree that a confidence interval not including 1.0 signifies statistical significance. However, the more commonly used statistical marker for significance is the p-value. As the p values are reported in the tables, it would make more sense to refer to it in the text as well.

Authors' response
We have included p values to all tables and added then to the text (abstract and results) when reporting statistical associations for the main results. We have not added P values to the text describing period stratification as differences will be driven by variation in statistical power. We have added the following to the methods section:
"…and used Cox proportional hazards regression to estimate hazard ratios with 95% CI and p-values."

Results:
11. - The sentence: Those admitted to hospital were more likely to have preexisting poorer health, as indicated by the Charlson Comorbidity Index and demyelinating disease prior to 2020" is confusing. Do you mean that a prior diagnosis of demyelinating disease is a risk factor for severe COVID-19? Then, in the following sentence, you say that all prior demyelinating diseases were excluded. Please clarify/rephrase.

Authors' response
Thank you for pointing out this error. The text is now:
"Those admitted to hospital were more likely to have pre-existing poorer health, as indicated by the Charlson Comorbidity Index. People with any demyelinating disease before baseline were excluded from all subsequent analysis."

12. - Please add p-values.

Authors' response

We have added p values to the text reporting statistical associations as described for point 10.

13. - What do you mean by "the outcomes were rare" (page #10)?

Authors' response
We have edited the sentence to read:
"There was only a small number of individuals with non-MS demyelinating disease outcomes associated with hospital admission for COVID-19 (n=12)."

14. - The list of non-MS demyelinating diseases other than neuromyelitis optica is vague and unclear (page #10).

Authors' response
Please see the responses to questions 5 and 9.
We have edited the text on diagnostic categories in the table and results section text to:
"Those with a raised risk associated with COVID-19 hospital admission were in the following diagnostic categories: neuromyelitis optica; clinically isolated syndrome and other specified acute disseminated demyelination; central demyelination of the corpus callosum; myelin oligodendrocyte glycoprotein antibody disease and other specified demyelinating diseases of the CNS; and unspecified diseases of the central nervous system."

15. - The median age at MS diagnosis in patients with prior SARS-CoV-2 infection was 56.7 years, which is rather old compared to the usual age of MS diagnosis. It would be interesting to know whether these newly diagnosed MS cases were of the relapsing or the progressive form (which tends to occur in older age).

Authors' response
Unfortunately, we do not have access to MS disease course in this dataset. We have added the following statement to the discussion:
"An alternative explanation is that the MS disease process had already begun but was undiagnosed in these individuals. This interpretation is consistent with the average age of MS diagnosis that we observed: onset at over 50 years of age suggests that the disease process began some years earlier."

16. - The paragraph describing the stratified analysis is hard to read. I am also not sure how informative it is.

Authors' response
We have removed table 6 (as small numbers in some categories make interpretation difficult), reduced the stratified analysis paragraph in the results section so now only refers to stratification by period as reported in (old) table 7. We have reduced the text on stratification in the discussion, as it added little (although the sentences on

stratification by period remain).

Discussion:
17.- In my opinion, the Discussion is too long and hard to follow. I would try to focus on the main findings and explain them, possibly leaving out the part regarding EBV infection (which is interesting but does not serve the scope of this paper and is somewhat hypothetical).

Authors' response
The discussion has been edited for clarity and reduced in length. We have removed sections on EBV as an outcome and the paragraph about stratified analysis.

18. - It is unclear which non-MS demyelinating disease the authors refer to as a risk factor for subsequent MS (page #14, lines # 10-12). For example, NMO is not a risk factor for MS.

'Authors' response
As in the introduction, we have made it clear that we examined demyelinating disease as the outcome, rather than identifying MS risk factors. The resolution of our ICD codes meant that some specific diagnoses could not be identified (please see points 5, 9 and 14).

19. - In the limitations, please mention that MOGAD was not included as a diagnosis.

Authors' response
Please see the response to point 5. This limitation is included in the discussion.

20. General comment: please check the text for grammatical errors and typos (i.e., unnecessary "("marks, etc.).

Authors' response
We have edited the text to try to remove errors.

Reviewer: 2

Comments to the Author
In this study the authors sought to understand the potential relationship between COVID infection and subsequent demyelinating disease. This is an area of great interest, as in the context of a novel pathogen, subsequent long term inflammatory conditions are a concern. As the authors imply, there is already strong associative evidence linking MS with EBV, and so links with COVID and EBV reactivation are a natural area of interest, particularly where high quality records and linkage data is available.

21. It is interesting that the signal is seen around hospital admissions (taken as a proxy for infection severity, which is appropriate), but not with PCR positive cases. Was PCR only available in symptomatic cases? Data suggests that a substantial proportion of COVID infection is asymptomatic, even in the pre-vaccine era. Was this accounted for at all in the analysis?

Authors' response
Testing was most common in people who were symptomatic, but also available to people without symptoms, including for those concerned about risk or prior to international travel. PCR testing was not available at the beginning of the pandemic, but became used widely. Thus, while not perfect, the stratification by period of infection might identity differences in the tested population between the earlier and later periods. We have modified a sentence in the discussion as follows: "Many further individuals may have been infected but were not tested by PCR (however, testing during the major period of the study period was extensive, although there was limited testing at the beginning of the pandemic. As we divided the analysis into two periods of infection, this may have helped to identify changes in the PCR positive population, particularly those who were not admitted to hospital"

I do have some further concerns linked to this.

22. Firstly, as the authors state, the assumed lag time is short between COVID and subsequent MS. Given current understanding around prodromal MS, and lag between radiological and clinical onset in many, there may be insufficient time for this study to really unpick this relationship. Further, the relationship that is seen may be due to (early) ascertainment bias, with those treated in hospital more likely to undergo neuroimaging, and so be diagnosed earlier than they might otherwise have been. This would potentially be supported by the lack of signal with non-hospitalised patients. I think this point requires further discussion.

Authors' response
We agree with this point and have extended the discussion to further consider surveillance bias.

We have included the following statements in the discussion section: "…..it should be considered that the association might be explained by shared susceptibility or surveillance bias (medical management of one condition results in diagnosis of another)." (second sentence in the discussion).
And
"Importantly, the results may have been influenced by surveillance/referral bias, as continued contact with healthcare following hospital admission for COVID-19 may have resulted in diagnosis of demyelinating disease."

The conclusion of the discussion reads: "We found an increased risk of demyelinating diseases of the CNS among people treated in hospital for COVID-19. A proportion of these associations with COVID-19 may potentially be due to shared susceptibility or surveillance bias."

23. Second, the number with inflammatory CNS disease following hospital admission with COVID is small - 12 in total, 7 of whom have unspecified neuroinflammation. I think that the text of the paper needs to be clearer regarding the small number and non-specific diagnoses that the positive conclusions are based on.

Authors' response

We have further clarified these points in the discussion. The number with CNS disease is notably larger if MS is included but, as we speculate in the paper, it is possible that the mechanism may be different. We have added the following to the discussion:
"The number of individuals with the outcome diagnoses of non-MS demyelinating diseases is small, and more than half of them have a non-specific diagnosis, which may question diagnostic accuracy so the results should be interpreted with caution. The Swedish ICD codes for the outcome diagnoses were limited to one digit after the decimal point, so some specific diagnoses were grouped. While this was not a problem for identification of MS, other groups contained diagnoses which would have been interesting to identify separately, such as clinically isolated syndrome, which is associated with future MS risk,[31] and myelin oligodendrocyte glycoprotein antibody disease, which is a separate disease entity.[32]"

24. I found the data around infectious mononucleosis interesting, however this felt a little disjointed from the inflammatory neurological diseases. There are challenges with healthcare-records reported IM, with relatively poor sensitivity and specificity overall. I am concerned that the symptoms of IM and "long COVID" may have substantial overlap and so recording bias may have driven this finding. Was IM evaluated using monospot or other laboratory test in any/some/all cases? This needs to be considered and discussed.

Authors' response
As suggested by referee 1, we have removed the sections on IM as an outcome from the manuscript (the sections on IM as possible mediating mechanism remain, even though this did not explain the associations).

25. More minor comments:
Whilst date is controlled for I could not see that vaccination status was considered? Is this data available and should it be included in the analysis?

Authors' response

As this would involve modelling another time-dependent covariates in such a large study population (particularly taking booster vaccinations into account) with relatively sparse outcomes this is problematic for convergence of the statistical models. While not a perfect solution, dividing the exposure before or after 1st January 2021 should be informative, as vaccination against SARS-CoV-2 began towards the end of 2020 in Sweden.

26. I think the abstract should be toned down in terms of findings, as it was not immediately clear that the positive findings related to hospital admissions only (and the small numbers) rather than COVID infection overall.

Authors' response

We have modified the abstract as suggested with the statements:
"Hospital admission for COVID-19 was associated with raised risk of subsequent non-multiple sclerosis demyelinating disease, but only 12 individuals had this

outcome among the exposed and of those seven has an unspecified demyelinating disease diagnosis."
And
"Equivalent associations with multiple sclerosis (28 individuals had this outcome among the exposed) were…."

27. Even though the timescale of the study is short, I wonder if survival curves showing time from infection to diagnosis of neuroinflammatory disease might help the reader.

Authors' response
We have created Kaplan–Meier plots as suggested. As the exposure was a time-dependent variable, the plot only shows events (diagnoses) and there is no reference line for the unexposed. We have added the following to the paper.

Statistical analysis:
"Kaplan–Meier plots were used to present the duration between hospital admission for COVID-19 and diagnosis of non-MS demyelinating disease and MS. "

Results:
"Figure 1 (panel A) shows the duration between hospital admission for COVID-19 and non-MS demyelinating disease diagnoses. The majority of these diagnoses were made in the first six months, but one third of them were diagnosed over a year after admission for COVID-19. "

"Figure 1 (panel B) shows the duration between hospital admission for COVID-19 and first MS diagnosis. The majority of these diagnoses were made in the first six months, and only approximately one fifth were diagnosed over a year after admission for COVID-19."

Discussion:
"The majority of diagnoses of both outcomes occurred during the first six months after hospital admission for COVID-19, suggesting precipitation of a pre-existing disease process or diagnoses made due to surveillance resulting from increased contact with healthcare professionals. A minority of the demyelinating disease diagnoses were made over a year after hospital admission for COVID-19, which does not eliminate the possibility of surveillance bias due to continued increased healthcare contact, but also suggests the importance of longer-term follow-up to identify associations with delayed disease onset."

Comments from the Scientific Editorial team:

-Please add an Abbreviated Summary (up to 50 words) of your paper that captures the main purpose and conclusions of the work. The summary should state the main results written in the third person ('[1st author surname] et al. report that …') and conclusion. This summary will be used in the contents list online and should not contain any abbreviations other than those on the accepted abbreviations list and accepted gene/protein names, making it accessible to the non-specialist. Please

upload it as a Word document. Allowed abbreviations are: AIDS; ANOVA; AMPA; ATP; A,T,C,G; CNS; CSF; CT; DNA; ECG; EEG; EMG; GABA; HIV; MRI; NMDA; PET; PCR; RNA.

Authors' response
This has been added.

-Please provide a graphical abstract. A graphical abstract is a visual representation of the central finding or methodology of the paper. It will be displayed in the table of contents. Authors should strive to make the graphical abstract informative, interesting, visually appealing, and straightforward (no caption needed, no abbreviations except gene names). The technical requirements for the graphical abstracts are:
- Size: 120 x 120 mm (300-600dpi) or 3000 x 3000 pixels
- Font: Arial 12-16 pt
- File type: tiff or PDF
- Aspect ratio: 1:1 preferred

Authors' response
This is now included

-Thank you for providing a data availability statement. Please also provide the codes generated and used in this work within the supplementary material or through a repository, of which the link has to be made available within the data availability statement.

Authors' response
This is provided.

-Tables 4, 5, 6: These tables are likely to exceed one journal page. Large tables that are more than one page in length, particularly in landscape orientation, are difficult to read and are not usually allowed; such tables should be uploaded as Supplementary material. Supplementary tables containing large amounts of text can be summarized in a smaller table for publication in the article if appropriate. Alternatively, you could consider splitting a large table into smaller tables. Please use the attached template to format your tables, and to check whether they are likely to exceed one typeset journal page.

Authors' response
We have dropped some of the tables, as requested by the referees. Only table 1 is reasonably large and it looks as if it should fit on one A4 page.
