# Supplementary material for: SARS-CoV-2 infection and risk of subsequent demyelinating diseases: national register–based cohort study
Source: Brain Commun. 2024 Nov 29;6(6):fcae406. doi: 10.1093/braincomms/fcae406 (PMC11629974; doi:10.1093/braincomms/fcae406)
Supplement: fcae406_Supplementary_Data [file Revision_1_ Response_to_reviewers_comments.pdf › Revision_1_ Response_to_reviewers_comments.pdf]

Revision One: Response to reviewers' comments

Dear Editor,

Please consider our revised paper "SARS-CoV-2 infection and risk of subsequent demyelinating diseases: national register-based cohort study" for publication in Brain Communications as an original article.

We have addressed each of the new comments made by the referee and editorial office, resulting, hopefully, to improvements. We have provided our point-by-point responses in the attached file.

Thank you for reconsidering our paper.

Yours faithfully

Scott Montgomery

Response to reviewers and editorial office
Reviewer(s)' Comments to Author:

Reviewer: 1

Comments to the Author

Thank you for revising the manuscript and addressing my comments. I believe the manuscript is improved and reads better. I have carefully read through the revised version and have the following comments:

Abstract:

1. -The sentence "Hospital admission for COVID-19 was associated with a subsequent raised risk of demyelinating diseases of the central nervous system" (lines 49-52) seems misplaced.

Authors' response

The sentence "Hospital admission for…" was intended as a concluding sentence. We have now removed it and altered the single remaining concluding sentence to: "Only a small number of non-MS demyelinating disease diagnoses were associated with hospital admission for COVID-19, and while the number with MS was somewhat higher, longer duration of follow-up will assist in identifying whether the associations are causal or due to shared susceptibility or surveillance bias, as these diseases can have long asymptomatic and prodromal phases."

2. -"Cox regression assessed the risk of two separate outcomes: hospital diagnosed non multiple sclerosis demyelinating diseases of the central nervous system and multiple sclerosis." This is not entirely true, as you also analyzed the risk in nonhospital admitted individuals. In the results section, you mention "hospital outpatient or inpatient diagnoses of non-MS demyelinating disease and of MS."

Authors' response

Hospital outpatients receive specialised hospital care but are not admitted (as opposed to non-hospital treatment in primary care). Only hospital diagnoses of these diseases would be considered reliable. We have clarified the setting for diagnoses in the first sentence under Outcomes in the methods section of the paper to indicate primary care diagnoses are not included:
"The outcomes studied were hospital inpatient and outpatient (but not primary care) diagnoses of non-MS demyelinating disease and MS."

3. -I would also mention that the number of MS and non-MS diagnoses you observed was very small (28 and 12, respectively), so your conclusions should be interpreted cautiously.

Authors' response

The current version of the abstract (modified due to previous reviewers' comments)

already contains the following:
“Hospital admission for COVID-19 was associated with raised risk of subsequent non-multiple sclerosis demyelinating disease, but only 12 individuals had this outcome among the exposed, and of those, seven has an unspecified demyelinating disease diagnosis.”

And
“Equivalent associations with multiple sclerosis (28 individuals had this outcome among the exposed) were rates of….”
We have modified the final sentence of the abstract to highlight small numbers as follows.
“Only a small number of non-MS demyelinating disease diagnoses were associated with hospital admission for COVID-19, and while the number with MS was somewhat higher, longer duration of follow-up will assist in identifying whether the associations are causal or due to shared susceptibility or surveillance bias, as these diseases can have long asymptomatic and prodromal phases.”
*

Introduction:

4. The current version of the introduction has been consistently modified. It feels like the introduction is lacking important information regarding what is already known about the risk of MS following SARS-CoV-2 infection. Moreover, some more details on the other non-MS demyelinating diseases are missing. Please elaborate on these disease entities and the possible causative role of SARS-CoV-2 infection in their pathogenesis.

Authors’ response

We have added the following paragraph to the introduction, which includes some additional references.

“In excess of 30% of patients with more severe COVID-19 have CNS lesions[9] and a case-series study of three patients, including one meeting MS criteria, suggests the infection could be implicated in causing inflammatory demyelinating disease.[10] In addition to MS, there are two further categories of CNS demyelinating disease in the Swedish version of the International Classification of Diseases 10 (ICD-10). The first is `other acute disseminated demyelination' that includes neuromyelitis optica spectrum disorders which involve optic nerve inflammation, and clinically isolated syndrome which is a first neurological episode that may in represent onset of MS in some individuals. The second category is `other demyelinating diseases of the central nervous system' which includes diagnoses such as myelin oligodendrocyte glycoprotein antibody-associated disease (MOGAD) where the host immune system attacks myelin in the brain, optic nerve and spinal cord. The precise mechanisms underlying associations of SARS-CoV-2 infection with a range of demyelinating diseases of the CNS are undefined,[11] but likely to involve triggering of autoimmunity among susceptible hosts through immune-mediated responses to eliminate the virus.[12] Mechanisms may include, the acute infection-related cytokine storm impairing myelination of neurons through an effect on glial cells;13 or renin–angiotensin–aldosterone system (RAAS) imbalance may fail to prevent immune

system-related damage to myelin;[11] and upregulation of a disintegrin and metalloprotease 17 (ADAM-17) limiting oligodendrocyte maturation and thus myelin formation.[11]"

Results:

5. Table 3 – the number of patients diagnosed with non-MS demyelinating disease with no prior SARS-COV-2 infection is 969 in this table, while it is 960 in Table 2. The number of patients diagnosed with non-MS demyelinating disease and positive SARS-CoV 2 test is 158 in this table, while it is 152 in Table 2. The sum of patients diagnosed with non-MS demyelinating disease with no prior SARS-COV-2 infection is 1010, while the total number mentioned in the first raw is 969. The sum of patients diagnosed with non-MS demyelinating disease and positive SARS-COV- 2 test is 165, while you say a total of 158. Please review these numbers and clarify the discrepancy.

Authors' response

Thank you very much for noticing this. Some migration influences on the population size were missed in descriptive statistics. This did not have any consequences for the results produced previously using Cox regression, as the those who migrated were censored appropriately. We have updated all tables to show the correct numbers of individuals. We have corrected the abstract and modified the first sentence of the materials and methods section:
"This register-based study included all individuals aged between three and 100 years who were resident in Sweden on 1st of January 2020 and after exclusion of those with any demyelinating disease, including MS, (n=21 538) a total of 9 959 818) were included in the analysis."

Discussion:

6. -" …An alternative explanation is that the MS disease process had already begun but was undiagnosed in these individuals. This interpretation is consistent with the average age of MS diagnosis that we observed: onset at over 50 years of age suggests that the disease process began some years earlier. The infection may have resulted in an exacerbation and disease progression, such that symptomatic onset led to an MS diagnosis…". I think you should make a more apparent distinction between disease onset and disease exacerbation here and propose a better explanation for your finding of old age of onset. Yes, MS pathogenesis may begin some years before the disease is clinically diagnosed, but this is true for MS cases diagnosed before the pandemic as well, and the typical age of diagnosis is still earlier. Why would the age of diagnosis in the post-pandemic era be older? Are you trying to make the point that COVID-19 is somehow associated with an older age of MS diagnosis compared to other infections?

Authors' response

We have clarified the text to make the point that it is possible that the infection caused worsening of subclinical disease, and this resulted in an MS diagnosis. The speculation about age at diagnosis in these patients has been removed, so the text

reads as follows.

"An alternative explanation is that the subclinical MS disease process had already begun but was previously undiagnosed in these individuals. The infection may have resulted in more rapid disease progression such that MS disease activity was no longer subclinical, leading to a diagnosis: both bacterial and viral infections are linked with MS exacerbations and disease progression.[23]"

7. -" One potential explanation of our findings of raised risks for demyelinating disease is that immune system perturbation is more common after SARS-CoV-2 infection resulting in greater risk of infectious mononucleosis due to Epstein-Barr virus, which is one of the most important environmental risk factors for MS. Our results do not support this explanation…" I still don't see why you chose to focus on EBV infection. Yes, it has been suggested as a risk factor for MS. But your study didn't find a case of EBV infection. I think this entire paragraph is confusing, and I would suggest omitting it.

Authors' response

We have now removed all sections of the manuscript on EBV/IM as a possible mediating factor and no longer speculate about potentially shared molecular mechanisms for EBV and SARS-CoV-2.

8. -" It is of note that non-MS diagnosis of demyelination diagnoses following hospital admission for COVID-19 tended to occur in earlier adulthood. The majority of diagnoses of both outcomes occurred during the first six months after hospital admission for COVID-19, suggesting precipitation of a pre-existing disease process…". I would suggest using a lower-toned phrasing here. While this may be possible, it is also possible that the pathogenic process that culminated in disease onset has taken place over a short period of time after the infection.

Authors' response

As suggested, we have modified the text as follows.
"The majority of diagnoses of both outcomes occurred during the first six months after hospital admission for COVID-19, which could indicate rapid onset of demyelinating disease, precipitation of a pre-existing disease process or diagnoses made due to surveillance bias resulting from increased contact with healthcare professionals."

Reviewer: 2

Comments to the Author

My comments have been addressed and I am happy with the revised manuscript
Comments from the editorial office:

9. For figure 1 please include a figure legend, which should include:
-Please provide details of the statistical analysis performed, the experimental unit(n=

mice,cells, wells, plate downs, etc)) and the number of data-points for each group analysed in these plots, and if the number is <10show the single data-points. Please note our policy on pseudo replication, we do not accept analyses where individual cells are the experimental unit when there is likely biological variability between animals, culture replicates, etc. Please see instructions for authors for more details.

For this type of data, we require either that a mean/median be taken per animal/culture replicate followed by statistical analyses or that more sophisticated analyses are performed such as mixed effects modelling which can account for random variables

Authors' response

This has been provided.

10. -Thank you for providing a data availability statement. Please also provide the codes generated and used in this work within the supplementary material or through a repository, of which the link has to be made available within the data availability statement.

Authors' response

The following has been added to the data availability statement.

"The codes generated and used in this work are available online as supplemental material."

11. -Tables 4, 5, 6: These tables are likely to exceed one journal page. Large tables that are more than one page in length, particularly in landscape orientation, are difficult to read and are not usually allowed; such tables should be uploaded as Supplementary material. Supplementary tables containing large amounts of text can be summarized in a smaller table for publication in the article if appropriate. Alternatively, you could consider splitting a large table into smaller tables. Please use the attached template to format your tables, and to check whether they are likely to exceed one typeset journal page.

-As a reminder at this revision stage, if you plan to edit the current figures and tables, or to make new figures and tables, please ensure that these items fit a typeset journal page (A4), or are uploaded as supplementary material.

Authors' response

All of the tables have been recreated so they fit an A4 page.
