# Supplementary material for: SARS-CoV-2 infection and risk of subsequent demyelinating diseases: national register–based cohort study
Source: Brain Commun. 2024 Nov 29;6(6):fcae406. doi: 10.1093/braincomms/fcae406 (PMC11629974; doi:10.1093/braincomms/fcae406)
Supplement: fcae406_Supplementary_Data [file Revision_1_decision_letter.pdf › Revision_1_decision_letter.pdf]

Revision One: Decision Letter

4th July 2024

Dear Dr. Montgomery,

Thank you for your recent submission BRAINCOM-2024-038.R1
entitled "SARS-CoV-2 infection and risk of subsequent demyelinating diseases: national register-based cohort study" to Brain Communications.

Your manuscript has been reviewed, and while the reviewers had largely positive comments, they have some constructive critiques that I think you could address in a minor revision of the manuscript. Therefore, I invite you to respond to the reviewer(s)' comments and revise your manuscript. The reviewer(s)' comments are included at the bottom of this message.

In addition to the reviewers' comments, please also address our Scientific Editor's comments added below to comply with our requirements for presentation of scientific data at Brain Communications.

Please format your revised manuscript according to our Instructions to Authors
https://academic.oup.com/braincomms/pages/General_Instructions
A summary of key points is attached to help with this. Please note that there can be substantial delays to publication if you don't resubmit in line with our requirements.

To revise your manuscript, click the link below or log into https://mc.manuscriptcentral.com/braincom and enter your Author Centre, where you will find your manuscript title listed under "Manuscripts with Decisions." Under "Actions," click on "Create a Revision." Your manuscript number will be appended to denote a new revision.

*** PLEASE NOTE: This is a two-step process. After clicking on the link, you will be directed to a webpage to confirm. ***

https://mc.manuscriptcentral.com/braincom?URL_MASK=1be3ff8dec2e493aa38db15ae1eec1db

Please upload a revised manuscript with changes tracked using track changes or in coloured text.

When submitting your revised manuscript, you will be asked to respond to the reviewer(s) comments. Please be as specific as possible in your response to all of the comments.

The editorial team at Brain Communications also aims to increase transparency in scientific publishing and to demonstrate the value that peer review brings to the scientific process. Please therefore consider whether you are willing to have reviewer comments on your paper and your response published as supplementary data with the paper if it is accepted. This is entirely optional and will only be done if both the authors and reviewers agree. When submitting your revision you will have the opportunity to update the answer you entered previously.

IMPORTANT: Your original files are available to you when you upload your revised manuscript. Please delete any redundant files before completing the submission.

Because we are trying to facilitate timely publication of manuscripts submitted to Brain Communications, we would appreciate your revised manuscript as soon as possible. If it is not possible for you to resubmit within 3 months, the option to submit a revision will expire, and we would then consider your revised paper as a new submission. However, if you need a little more time, please get in touch and we can extend this deadline for you.

Please note that Brain Communications is a fully open access journal, and if your article is accepted for publication, an open access charge will be payable (this does not apply to letters and scientific commentaries). Details can be found in our Instructions for Authors: https://academic.oup.com/braincomms/pages/General_Instructions

Once again, thank you for submitting your manuscript to Brain Communications and I look forward to receiving your revision.

Best wishes

Dr. Josef Priller
Associate Editor, Brain Communications

Professor Tara Spires-Jones
Founding Editor, Brain Communications

Reviewer(s)' Comments to Author:

Reviewer: 1

Comments to the Author
Thank you for revising the manuscript and addressing my comments. I believe the manuscript is improved and reads better. I have carefully read through the revised version and have the following comments:

Abstract:

-The sentence “Hospital admission for COVID-19 was associated with a subsequent raised risk of demyelinating diseases of the central nervous system” (lines 49-52) seems misplaced.

-“Cox regression assessed the risk of two separate outcomes: hospital diagnosed non multiple sclerosis demyelinating diseases of the central nervous system and multiple sclerosis.” This is not entirely true, as you also analyzed the risk in non-hospital admitted individuals. In the results section, you mention “hospital outpatient or inpatient diagnoses of non-MS demyelinating disease and of MS.”

-I would also mention that the number of MS and non-MS diagnoses you observed was very small (28 and 12, respectively), so your conclusions should be interpreted cautiously.

Introduction:
The current version of the introduction has been consistently modified. It feels like the introduction is lacking important information regarding what is already known about the risk of MS following SARS-CoV-2 infection. Moreover, some more details on the other non-MS demyelinating diseases are missing. Please elaborate on these disease entities and the possible causative role of SARS-CoV-2 infection in their pathogenesis.

Results:

Table 3 – the number of patients diagnosed with non-MS demyelinating disease with no prior SARS-COV-2 infection is 969 in this table, while it is 960 in Table 2. The number of patients diagnosed with non-MS demyelinating disease and positive SARS-CoV 2 test is 158 in this table, while it is 152 in Table 2. The sum of patients diagnosed with non-MS demyelinating disease with no prior SARS-COV-2 infection is 1010, while the total number mentioned in the first raw is 969. The sum of patients diagnosed with non-MS demyelinating disease and positive SARS-COV- 2 test is 165, while you say a total of

158. Please review these numbers and clarify the discrepancy.

Discussion:

-" ...An alternative explanation is that the MS disease process had already begun but was undiagnosed in these individuals. This interpretation is consistent with the average age of MS diagnosis that we observed: onset at over 50 years of age suggests that the disease process began some years earlier. The infection may have resulted in an exacerbation and disease progression, such that symptomatic onset led to an MS diagnosis...". I think you should make a more apparent distinction between disease onset and disease exacerbation here and propose a better explanation for your finding of old age of onset. Yes, MS pathogenesis may begin some years before the disease is clinically diagnosed, but this is true for MS cases diagnosed before the pandemic as well, and the typical age of diagnosis is still earlier. Why would the age of diagnosis in the post-pandemic era be older? Are you trying to make the point that COVID-19 is somehow associated with an older age of MS diagnosis compared to other infections?

-" One potential explanation of our findings of raised risks for demyelinating disease is that immune system perturbation is more common after SARS-CoV-2 infection resulting in greater risk of infectious mononucleosis due to Epstein-Barr virus, which is one of the most important environmental risk factors for MS. Our results do not support this explanation..." I still don't see why you chose to focus on EBV infection. Yes, it has been suggested as a risk factor for MS. But your study didn't find a case of EBV infection. I think this entire paragraph is confusing, and I would suggest omitting it.

-" It is of note that non-MS diagnosis of demyelination diagnoses following hospital admission for COVID-19 tended to occur in earlier adulthood. The majority of diagnoses of both outcomes occurred during the first six months after hospital admission for COVID-19, suggesting precipitation of a pre-existing disease process...". I would suggest using a lower-toned phrasing here. While this may be possible, it is also possible that the pathogenic process that culminated in disease onset has taken place over a short period of time after the infection.

Reviewer: 2

Comments to the Author
My comments have been addressed and I am happy with the revised manuscript

Comments from the editorial office:

For figure 1 please include a figure legend, which should include:

-Please provide details of the statistical analysis performed, the experimental unit(n= mice,cells, wells, plate downs, etc)) and the number of data-points for each group analysed in these plots, and if the number is <10show the single data-points. Please note our policy on pseudo replication, we do not accept analyses where individual cells are the experimental unit when there is likely biological variability between animals, culture replicates, etc. Please see instructions for authors for more details. For this type of data, we require either that a mean/median be taken per animal/culture replicate followed by statistical analyses or that more sophisticated analyses are performed such as mixed effects modelling which can account for random variables

-Thank you for providing a data availability statement. Please also provide the codes generated and used in this work within the supplementary material or through a repository, of which the link has to be made available within the data availability statement.

-Tables 4, 5, 6: These tables are likely to exceed one journal page. Large tables that are more than one page in length, particularly in landscape orientation, are difficult to read and are not usually allowed; such tables should be uploaded as Supplementary material. Supplementary tables containing large amounts of text can be summarized in a smaller table for publication in the article if appropriate. Alternatively, you could consider splitting a large table into smaller tables. Please use the attached template to format your tables, and to check whether they are likely to exceed one typeset journal page.

-As a reminder at this revision stage, if you plan to edit the current figures and tables, or to make new figures and tables, please ensure that these items fit a typeset journal page (A4), or are uploaded as supplementary material.
