# Supplementary material for: SARS-CoV-2 infection and risk of subsequent demyelinating diseases: national register–based cohort study
Source: Brain Commun. 2024 Nov 29;6(6):fcae406. doi: 10.1093/braincomms/fcae406 (PMC11629974; doi:10.1093/braincomms/fcae406)
Supplement: fcae406_Supplementary_Data [file Revision_2_ Response_to_reviewers_comments.pdf › Revision_2_ Response_to_reviewers_comments.pdf]

Revision 2: Response to reviewers' comments

Dear Editor,

We have revised our paper "SARS-CoV-2 infection and risk of subsequent demyelinating diseases: national register-based cohort study" for publication in Brain Communications as an original article, to address the comment from the scientific editorial office (add A and B to the panels of the figure). The main manuscript text is unaltered, except for the addition of the figure legend. We have also corrected a typing error in the title of the second column in table 4 (MS n/ total n).

Thank you for the suggestions to improve our paper.

Yours faithfully

Scott Montgomery
